# The effects of reduced nicotine content cigarettes on biomarkers of nicotine and toxicant exposure, smoking behavior and psychiatric symptoms in smokers with mood or anxiety disorders: A double-blind randomized trial

**DOI:** 10.1371/journal.pone.0275522

**Published:** 2022-11-02

**Authors:** Jonathan Foulds, Susan Veldheer, Gladys Pachas, Shari Hrabovsky, Ahmad Hameed, Sophia I. Allen, Corinne Cather, Nour Azzouz, Jessica Yingst, Erin Hammett, Jennifer Modesto, Nicolle M. Krebs, Courtney Lester, Neil Trushin, Lisa Reinhart, Emily Wasserman, Junjia Zhu, Jason Liao, Joshua E. Muscat, John P. Richie, A. Eden Evins

**Affiliations:** 1 Penn State Center for Research on Tobacco and Health, Department of Public Health Sciences, Pennsylvania State University—College of Medicine, University Drive, Hershey, PA, United States of America; 2 Department of Family and Community Medicine, Penn State College of Medicine, Hershey, PA, United States of America; 3 Center for Addiction Medicine, Department of Psychiatry, Massachusetts General Hospital, Boston, MA, United States of America; 4 Harvard Medical School, Boston, MA, United States of America; 5 Ross and Carol Nese College of Nursing, Penn State University, State College, PA, United States of America; 6 Department of Psychiatry, Pennsylvania State University—College of Medicine, Hershey, PA, United States of America; University College London, UNITED KINGDOM

## Abstract

**Background:**

The U.S. Food and Drug Administration and the government of New Zealand have proposed a reduction of the nicotine content in cigarettes to very low levels. This study examined the potential effects of this regulation in smokers with affective disorders.

**Methods:**

In a randomized controlled parallel group trial conducted at two sites in the USA (Penn State University, Hershey, PA and Massachusetts General Hospital, Boston, MA) 188 adult smokers with a current (n = 118) or lifetime (n = 70) anxiety or unipolar mood disorder, not planning to quit in the next 6 months, were randomly assigned (1:1) to smoke either Usual Nicotine Content (UNC) (11.6 mg nicotine/cigarette) research cigarettes, or Reduced Nicotine Content (RNC) research cigarettes where the nicotine content per cigarette was progressively reduced to 0.2 mg in five steps over 18 weeks. Participants were then offered the choice to either receive assistance to quit smoking, receive free research cigarettes, or resume using their own cigarette brand during a 12-week follow-up period. Main outcomes were biomarkers of nicotine and toxicant exposure, smoking behavior and dependence and severity of psychiatric symptoms. The pre-registered primary outcome was plasma cotinine.

**Results:**

A total of 143 (76.1%) randomized participants completed the randomized phase of the trial, 69 (73.4%) in the RNC group and 74 (78.8%) in the UNC group. After switching to the lowest nicotine content cigarettes, compared to smokers in the UNC group, at the last randomized visit the RNC group had significantly lower plasma cotinine (metabolite of nicotine): difference between groups, -175.7, 95% CI [-218.3, -133.1] ng/ml. Urine NNAL (metabolite of NNK, a lung carcinogen), exhaled carbon-monoxide, cigarette consumption, and cigarette dependence were also significantly lower in the RNC group than the UNC group. No between-group differences were found on a range of other biomarkers (e.g. 8-isoprostanes) or health indicators (e.g. blood pressure), or on 5 different psychiatric questionnaires, including the Kessler K6 measure of psychological distress. At the end of the subsequent 12-week treatment choice phase, those randomized to the RNC group were more likely to have quit smoking, based on initial intent-to-treat sample, n = 188 (18.1% RNC v 4.3% UNC, p = 0.004).

**Conclusion:**

Reducing nicotine content in cigarettes to very low levels reduces some toxicant exposures and cigarette addiction and increases smoking cessation in smokers with mood and/or anxiety disorders, without worsening mental health.

**Trial registration:**

TRN: NCT01928758, registered August 21, 2013.

## Introduction

Tobacco smoking remains the leading preventable cause of premature morbidity and mortality in the U.S. [1]. The U.S. Food and Drug Administration (FDA) regulates tobacco products, and recently reintroduced plans to reduce the nicotine content of cigarettes to minimally addictive levels [2, 3].While these plans remain under consideration in USA, the government of New Zealand recently announced a plan to allow only reduced nicotine cigarettes to be sold [4]. Previous studies have generally found that reduction of nicotine content in cigarettes is feasible and safe in smokers with and without comorbid psychiatric illness, and it has been estimated that this would save millions of lives [5–15]. Over 25% of smokers have an affective (unipolar mood or anxiety) disorder, representing over 8 million people in the US. Affective disorder smokers (ADS) report more severe nicotine withdrawal symptoms and lower success rates when attempting cessation [16–19]. It has been speculated that a policy to reduce the nicotine content in cigarettes may have the unintended consequences, particularly in vulnerable subgroups such as ADS, of exacerbating psychiatric symptoms or causing compensatory heavier smoking that could increase their exposure to toxicants in tobacco smoke [20, 21]. Indeed, in one recent trial [13] among smokers with mood disorders, those randomized to very low nicotine cigarettes had significantly higher mean Beck Depression Inventory scores during the trial than those randomized to normal nicotine cigarettes. One of the largest randomized trials of reduced nicotine cigarettes in a non-psychiatric population [12], found that 20 weeks after randomization to very low nicotine cigarettes, 7% had quit smoking, as compared to 2% of those randomized to smoke regular nicotine cigarettes. This suggests that if ADS can tolerate reduced nicotine cigarettes (RNC) without psychiatric symptom exacerbation, reduced severity of nicotine dependence on RNC may improve smoking cessation rates in this population. This study examined the effects of reduced nicotine content cigarettes on psychiatric symptoms, severity of dependence, toxicant exposure and early abstinence rates in ADS. The primary hypothesis was that smokers assigned RNC cigarettes would have lower plasma cotinine than those assigned Usual Nicotine Content cigarettes at the end of the randomized phase.

## Methods

Detailed methods and design of this two-site, two-arm, double-blind, parallel group, randomized controlled 33-week trial have been previously reported [22], and both the original protocol (09/2015) and final protocols are outlined in S1and S2 Files, but are summarized here. This study was approved by the Penn State Hershey and Massachusetts General Hospital Institutional Review Boards. Participants were recruited at both sites throughout the Hershey and Boston areas by using media advertisements (newspaper, radio, internet); study posters and flyers placed on community message boards, in local businesses, and in clinics; community newsletters, social media sites (e.g., Facebook) and internet websites (e.g., Craigslist). Interested volunteers who called the study centers first completed basic eligibility questions over the phone. After meeting eligibility criteria over the phone, participants were scheduled to come into the study center where they were consented to the study and further screened and assessed for eligibility.

### Study population

Participants were 188 adults, who met the following inclusion and exclusion criteria prior to starting the study: **Inclusion criteria**: aged 18–65, planning to live in the local area for the next 8 months, report smoking >4 cigarettes per day (regular filtered cigarettes or machine-rolled cigarettes with a filter) for at least the past 12 months, no quit attempt in the prior month and not planning to quit smoking in the next 6 months, no use of varenicline, bupropion (used specifically as a cessation aid); nicotine patch; gum; lozenge; inhaler; or nasal spray in prior month, meet lifetime diagnostic criteria for a current or lifetime unipolar mood disorder (dysthymia, major or minor depression, premenstrual dysphoric disorder) or anxiety disorder (panic disorder, obsessive-compulsive disorder; post-traumatic stress disorder; mixed anxiety depressive disorder, agoraphobia, generalized anxiety disorder, social phobia, specific phobia) based on the Mini-International Neuropsychiatric Interview (MINI) [23], and ability to read and write in English. **Exclusion criteria**: pregnant and/or nursing, any unstable or significant medical conditions such as elevated blood pressure (systolic >160 mmHg at baseline), recent heart attack or some other heart condition, stroke, or severe angina, COPD requiring oxygen, use of oral prednisone, kidney disease (e.g., dialysis) or liver diseases (e.g., cirrhosis), any medical disorder/medication that may affect participant safety or biomarker data, use of any non-cigarette nicotine delivery product (e.g., cigar, pipe, chew, snus, dip, hookah, electronic cigarette, strips, sticks) in the past 7 days, other serious mental illness (e.g., schizophrenia, bipolar disorder, current eating disorder, or dementia) or any inpatient psychiatric or substance abuse treatment in the past 6 months, current suicide risk on clinical assessment (above “low risk” score on MINI [23] diagnostic interview), weekly use in the past 3 months of illegal drugs or prescription drugs that are not being used for medically prescribed purposes, alcohol use that would hinder the participant’s ability to participate, a history of difficulty providing or unwilling to provide blood samples (e.g., fainting, poor veins), surgery requiring general anesthesia in the past 6 weeks, unwilling to remain on one flavor of research cigarette (regular or menthol) for the duration of the trial or smokes hand-rolled cigarettes, another member of household currently participating in the study, prisoner (at the time of enrollment), any other condition or situation that would, in the investigator’s opinion, make it unlikely that the participant could adhere to the study protocol.

Recruitment occurred at Penn State Medical Center in Hershey, Pennsylvania (n = 100) and Massachusetts General Hospital in Boston, Massachusetts (n = 88), both in the U.S.A. All study data were collected and managed using REDCap (Research Electronic Data Capture) hosted at the Penn State College of Medicine [24].

### Procedures

During **Baseline I**, participants smoked their own brand of cigarettes for one week. At **Baseline II**, all participants were asked to use only SPECTRUM research cigarettes with a usual nicotine content (11.6 mg) for two weeks. From Baseline II and throughout the rest of the trial, research cigarettes were provided at no cost to participants. Participants were instructed to only smoke the provided research cigarettes, and to avoid use of other cigarettes, other tobacco products (including electronic cigarettes) and non-prescribed substances.

Participants who completed Baseline II and agreed to continue then entered the **Randomized Phase (III).** They were randomized (1:1 ratio) to either (1) continue to smoke the same 11.6 mg nicotine SPECTRUM research cigarettes they smoked in Baseline II for 18 additional weeks (UNC) or (2) switch to identical appearance cigarettes with progressively reduced nicotine content (RNC). Nicotine content in RNC cigarettes was reduced every 3 weeks over 18 weeks from 11.6 mg/cigarette to 0.2 mg/cigarette, remaining on this lowest level during the last 6 weeks of the randomized phase. In a prior pharmacokinetic study, a single UNC research cigarette provided a boost to plasma nicotine of 17.3 ng/ml, similar to an own-brand cigarette (19 ng/ml), whereas the 0.2mg nicotine RNC provided a nicotine boost of only 0.3 ng/ml [25].

#### Randomization

Participants were randomized 1:1 to reduced nicotine or usual nicotine cigarettes based on a predetermined random number sequence generated by the study statistician stratified by site (Penn State and Mass. General) and by preferred flavor (regular/menthol) with a block size of six.

#### Blinding

A Cigarette Management System was used to manage assigning randomized, blinded cigarettes to participants and to track cigarette inventory [26]. The Cigarette Management System was maintained within the Investigational Drug Pharmacy (IDP) at each site, and concealment was achieved by having IDP staff (with no participant contact) prepare cigarette cartons that were identical for each participant other than that each carton displayed a blind code. Only the study statistician and the IDP staff-member had access to the link between the blind code and the nicotine content information contained within the Cigarette Management System. This ensured that randomization was concealed and researchers and participants were blind to the randomized allocation throughout the trial. During the Randomized Phase, participants attended study visits and received research cigarettes every three weeks. They were provided with 150% of daily cigarette consumption reported at baseline to ensure they had an adequate supply to last until their next visit. Participants and study staff were blind to the experimental cigarette allocation throughout the randomized and treatment choice phases of the trial.

At the last visit (end of 18^th^ week) of the Randomized Phase, participants began the 12-week **Treatment Choice Phase (IV)**. Participants were given a copy of the U.S. Surgeon General Report, “How Tobacco Causes Disease” and were asked to choose one of the following options:

1. Return to smoking their usual brand of cigarettes for 12 weeks (at their own cost).
2. Continue to receive the same research cigarettes they were currently smoking (still double-blind) for a further 12 weeks (provided at no cost).
3. Quit smoking with brief counseling from the study team and the option to use oral nicotine replacement therapy (NRT [gum or lozenge]) for 11 weeks.

All participants were asked to attend two study visits in the Treatment Choice Phase at 4 and 12 weeks after the end of the randomized phase (visits 11 and 12).

The sequence for all study visits, a list of all measures at these study visits and a detailed list of the nicotine content dosing schedule are provided in S2 Fig and S1, S2 Tables in S3 File. As described in S1 Table in S3 File, participants were compensated between $40 and $80 for time and travel after each visit, $10 for phone surveys, plus $100 for completing all visits and returning over 80% of unused cigarette packs. The maximum total compensation was $1000.

### Assessments

Biomarkers of exposure included plasma cotinine [pre-registered primary outcome, measured at the end of the randomized phase], exhaled carbon monoxide, urinary total NNAL, GSSP:GSH (Ratio of Glutathione to Oxidized Glutathione) and 1-hydroxypyrene. See S3 File for detailed methodology for biomarker analyses. These biomarkers and self-report of cigarette consumption were assessed at baseline visit 2 and repeated visits through 18 weeks after randomization (visit 10). At each visit, participants were asked if they had smoked any non-research cigarettes or used any other nicotine products or marijuana. Psychiatric and nicotine withdrawal symptoms were assessed with the Quick Inventory of Depressive Symptomatology (QIDS [depression measure]) [27], Overall Anxiety Severity and Impairment Scale (OASIS [anxiety measure]) [28], PSS (Perceived Stress Scale) [29], CES-D (*Center for Epidemiological Studies-Depression* Scale) [30], Kessler K6 [31], QSU (Questionnaire on Smoking Urges-short form) [32], and Minnesota Nicotine Withdrawal Scale [33]. Assessments of tobacco dependence (Fagerstrom Test of Cigarette Dependence [34], HONC (Hooked on Nicotine Checklist) [35] and the Penn State Cigarette Dependence Index [36] were measured at each visit. Health status (e.g. pulse, blood pressure, body weight, waist to hip ratio) and respiratory health outcomes (e.g. lung function test [FEV1] [37] and Clinical COPD Questionnaire [CCQ] [38]) were measured during baseline and randomized phases. Adverse events were collected at every visit if participants had any new or worsening health symptoms, or had any reason to change their medication. Adverse events were classified using the Medical Dictionary for Regulatory Activities (MeDRA) coding system. Each symptom was rated for severity (mild, moderate, severe, life-threatening) and for likelihood of relationship to study participation (unrelated, unlikely, possibly, probably, or definitely related). Severe adverse events were classified as those that were life-threatening, required inpatient hospitalization, or resulted in persistent disruption of normal life functions. All adverse events were reviewed by a licensed physician. Self-report of intention to quit smoking, and actual smoking cessation were assessed at the treatment choice phase (visits 10–12, weeks 21–33). Abstinence at visits 11 and 12 was defined as self-report of no tobacco use in the prior 7 days, validated by exhaled CO<10ppm, using an intent-to-treat analysis based on all randomized participants, assuming dropouts to be continuing smokers.

### Sample size and statistical analysis

The plausible effect size and variation for the power calculation was based on the results of a similar study by Benowitz and colleagues [6] in which the mean and standard deviation of plasma cotinine were 240 and 120 for the control group and 113 and 116 for the reduced nicotine group at the week 22 follow-up (end of randomization phase for the trial). We anticipated the possibility of a smaller difference in means in our study. The study was powered to detect a between group difference in plasma cotinine concentration of 58 ng/ml with at least 80% power, and a difference of 68 ng/ml with at least 90% power, based on 100 participants per group being randomized, and 70 participants per group completing the randomized phase, based on two-tailed tests with an alpha level of 0.05. The prior trial [6] experienced differential dropout (9% v 33%). This trial had at least 90% power to detect a difference in dropout of that magnitude.

The statistical analysis focused on comparing the intervention and control groups, RNC vs. UNC, on (a) plasma cotinine concentration (primary outcome) at the end of the randomized phase (visit 10, 18 weeks after randomization); (b) secondary quantitative outcomes, e.g. exhaled CO, QIDS depression level and OASIS anxiety at the end of the randomized phase; (c) dropout rate during the randomized phase; (d) rate of psychiatric and other serious AEs and (e) the proportion of participants in each group choosing to try to quit and who quit smoking at the end of the treatment choice phase. Linear regression models were constructed for each quantitative outcome variable, for measures taken from the randomization visit through to the end of the randomized phase (visit 10). Unadjusted regression models compared the two trial arms while controlling for the baseline value of the outcome measure (recorded at visit 4). Adjusted models then evaluated the randomized treatment effect while adjusting for other baseline covariates that were selected via backward elimination using a significance level of 0.1. These models were built on data from subjects who completed the randomized phase and were intended to focus on comparing outcomes between those who had completed 6 weeks of smoking the lowest nicotine content cigarettes in the RNC group, with those in the UNC group at that same visit (v10). A separate analysis was also conducted for participants who reported exclusive, per protocol, use of assigned research cigarettes (“compliers”), biochemically validated for those smoking the lowest nicotine content cigarettes using plasma cotinine as previously reported [39]. This analysis aimed to focus on those who had not used any non-research cigarettes. Linear mixed-effect models for repeated measures were used to analyze the change over time (visits) in the main quantitative outcome measures. These analyses used data from all participants at all visits, regardless of dropout, and was intended as a sensitivity check on the main quantitative outcomes (i.e. checking that the pattern of results based on completers on the main quantitative outcomes [cotinine, cigarettes per day, exhaled CO, QIDS, OASIS, Kessler K6, and PSS], was the same as analyses including all participants). Chi-squared or Fisher’s Exact tests were was used to compare the intention to quit smoking (yes/no) at the end of the randomized phase (Visit 10), and abstinence in the treatment choice phase between the two groups. A Kaplan-Meier time-to-event analysis was used to compare the time from randomization to dropout between the two groups. R statistical software was used for analyses and statistical significance was assumed to be p<0.05 [40].

Intention-to-treat analyses, including all randomized participants, were used in some analyses (e.g. linear mixed-effects models and analyses of smoking cessation in the treatment choice phase). The main outcome analyses were based on data collected at the last randomized visit (visit 10) and so were necessarily based on completers. Additional sub-group analyses were carried out based on participants who completed the randomized phase and complied with the protocol requirement to only use supplied research cigarettes (per protocol analyses).

### Changes from original protocol

The basic trial methods remained consistent with the original protocol of 09/2015 with the following changes: (a) some of the originally proposed biomarkers were not measured, as they were replaced by a more relevant but similar biomarker (e.g. Phenanthrene Tetrol replaced by 1-hydroxypyrene) and others (e.g. 8-OHdG) because we found that the commercially available kits were not sufficiently reliable, (b) we originally proposed to randomize 200 participants, expecting 140 to complete the randomized phase, but due to issues with supply of research cigarettes we stopped recruitment with 188 randomized, of whom 143 completed the randomized phase, (c) we originally proposed to validate cigarette abstinence with exhaled CO<6ppm and cotinine <15ng/ml, but changed this to CO<10ppm because it was part of the original protocol to offer participants treatment with nicotine replacement therapy after the randomized phase which would increase cotinine levels, and in 2016 we became aware that the brand of CO monitor we were using (COvita by Bedfont) provided readings averaging 3.8ppm higher than the widely used Vitalograph monitors [41] (d) the original analysis plan stated that the primary outcome would be “plasma cotinine during the last 3 weeks of the randomized phase” and that “Differences between groups will be computed at each time point”. The primary analyses presented here are based on measures at the last visit (visit 10) of the randomized phase (covering the prior 3 weeks), and results for linear mixed effects models are also presented in the S3 File. Most of these changes, including the CO cut-point to verify cigarette abstinence, were included in our protocol paper published in 2017, prior to most of the data collection and prior to any data analysis [22].

## Results

**Fig 1:**
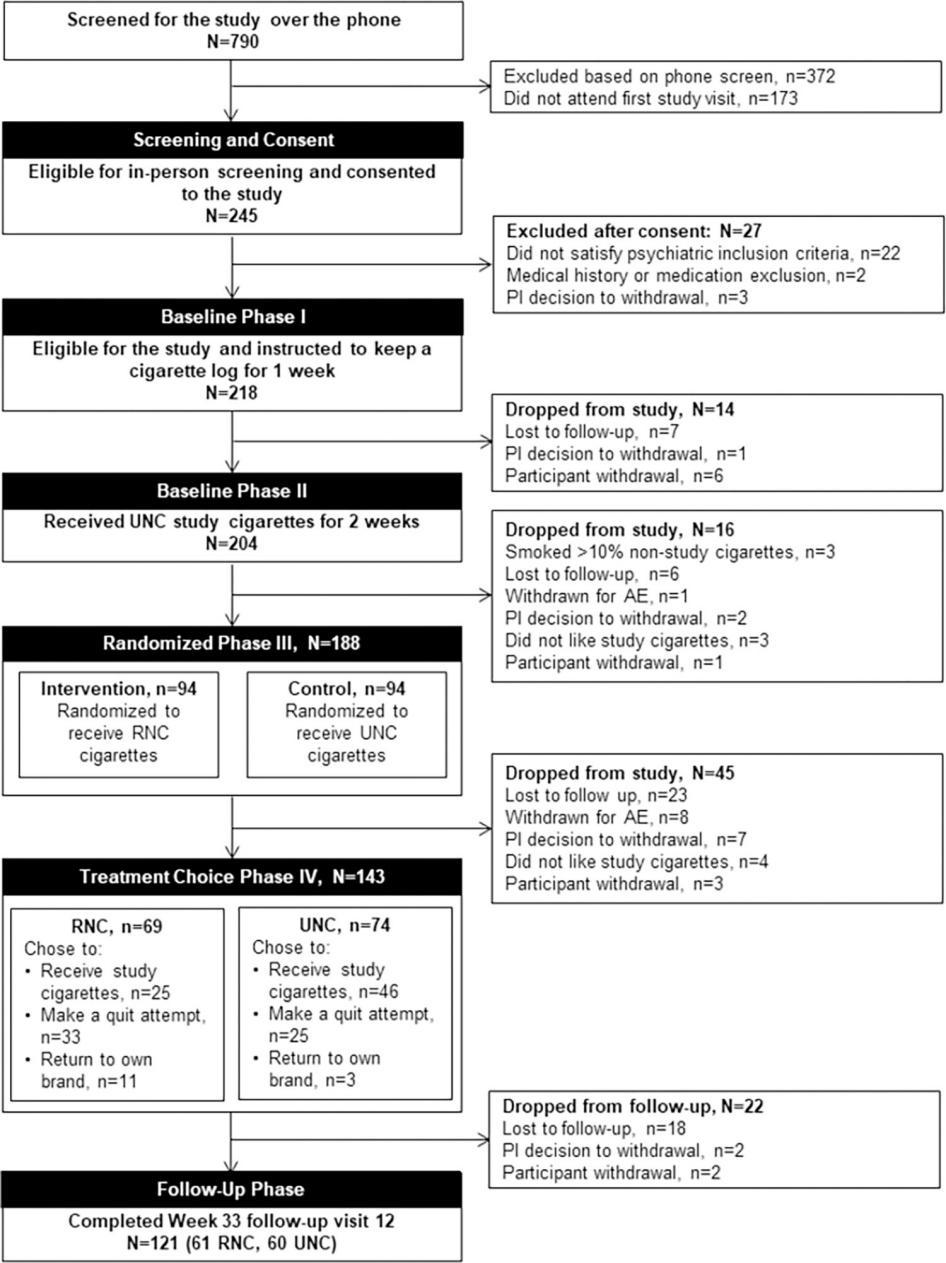
CONSORT participant flow diagram.

Participants were recruited between September 2015 and August 2017, and the last participant completed the study in March 2018. 790 potential participants were screened by telephone, of whom 372 were ineligible and 173 did not attend the first in-person visit. Of the 245 who attended visit 1, 27 did not satisfy psychiatric (22), medical (2) or other (3) inclusion criteria. 218 started Baseline I (smoking their own cigarettes for a week), of whom 7 were lost to follow-up, 6 withdrew themselves and one was withdrawn by the PI due to a Serious Adverse Event. 204 began Baseline II (smoking Usual Nicotine Content research cigarettes for two weeks), of whom 3 were withdrawn due to excessive use of non-research cigarettes, 6 were lost to follow-up, one was withdrawn due to an adverse event, one chose to withdraw and 3 withdrew because they did not like the research cigarettes. Fig 1 shows the CONSORT participant flow diagram for the trial. A total of 143/188 (76.1%) of randomized participants completed the randomized phase of the trial, 73.4% (69/94) for the RNC group and 78.8% (74/94) for the UNC group. A time-to-event analysis revealed no significant difference in time-to-dropout between the two arms (log-rank p = 0.41). Table 1 shows the baseline characteristics of the two randomized groups on key demographic, clinical and smoking history variables. The groups were well matched, and only one of these was significantly different at baseline: 60 (63%) of the UNC group had a current anxiety disorder diagnosis, compared to 45 (47.9%) of the RNC group (chi-squared test, p = 0.0395). Over 54% in both groups were taking psychiatric medications and over 57% met criteria for a current mood or anxiety disorder (the rest having past diagnoses).

**Table 1:** Study participant demographic and smoking characteristics pre-randomization.

	Reduced nicotine content (n = 94)	Usual nicotine content (n = 94)
Female, % (n)	63.8 (60)	57.4 (54)
Race, % (n)		
African American	16.0 (15)	11.7 (11)
White	74.5 (70)	80.9 (76)
Other	9.6 (9)	7.4 (7)
Age (in years), mean (SD, range)	43.3 (11.7, 21–65)	43.1 (13.3, 19–65)
Bachelor’s degree or higher, % (n)	21.3 (20)	20.2 (19)
Currently employed full-time, % (n)	37.6 (35) [n = 93]	38.0 (35) [n = 92]
Median number of prior attempts to quit smoking (% with no prior quit attempts)	2 (19.1)	2 (26.6)
Smoke menthol cigarettes, % (n)	40.4 (38)	38.3 (36)
Number of years as daily smoker, mean (SD, range)	25.4 (12.4, 2–49)	26.1 (13.4, 1–53)
Cigarettes per day, mean (SD, range)	18.7 (10.0, 5–60)	20.5 (10.0, 5–60)
Exhaled carbon monoxide (in ppm), mean (SD, range)	27.6 (17.0, 4–100)	27.7 (16.5, 6–85)
Moderate or higher environmental smoke exposure score, % (n)	69.1 (65)	74.2 (69) [n = 93]
Fagerström Test for Cigarette Dependence score, mean (SD, range)	5.8 (2.3, 0–10)	6.0 (2.2, 1–10)
CES-D* score, mean (SD, range)	18.2 (8.7, 4–43) [n = 92]	18.9 (7.7, 5–43) [n = 93]
Kessler K6 score, mean (SD, range)	5.8 (5.4, 0–22) [n = 93]	6.9 (5.3, 0–20)
Penn State Cigarette Dependence Index	12.9 (3.4, 5–20)	13.4 (3.4, 6–20)
score, mean (SD, range)	[n = 93]	
Lifetime suicidality, % (n)	34.0 (32)	29.8 (28)
Number of MINI mood/anxiety disorder		
diagnoses*, % (n)		
One current diagnosis	30.9 (29)	33.0 (31)
Two or more current diagnoses	26.6 (25)	35.1 (33)
Past diagnosis/-es only	42.6 (40)	31.9 (30)
Current/[Past] mood disorder, %	24.5 / [67.0]	27.7 / [63.8]
Current/[Past] anxiety disorder, %	47.9 / [48.9]	63.8 / [45.7]
Currently prescribed at least one medication for psychiatric reasons, % (n)	54.3 (51)	55.3(52)

*Abbreviations: CES-D (*Center for Epidemiological Studies-Depression* Scale), MINI (Mini-International Neuropsychiatric Interview). Gender, race, age, education, employment, quit attempts, menthol flavor preference, number of years as daily smoker, CES-D score, lifetime suicidality, number of MINI mood/anxiety disorder diagnoses, current/past anxiety/mood disorder, use of medication for psychiatric reasons assessed at Visit 1 at the time of study enrollment.

Cigarettes per day (Measured via self-report “How many cigarettes per day do you usually smoke?”), exhaled CO, environmental smoke exposure, FTCD, Kessler K6, PSCDI assessed at Visit 2 at the start of Baseline Phase I.

Baseline and end of randomized phase (v10) values for the primary outcome (plasma cotinine) is provided in Table 2, along with results of statistical comparisons based on linear regression models. This shows that at visit 10, those randomized the RNC group had plasma cotinine levels that were 175.7 ng/ml lower than those randomized to the UNC group (95% Confidence Interval -218.3, -133.1) after controlling for baseline plasma cotinine at visit 4. This difference remained statistically significant after controlling for other predictive baseline variables, and also in analyses only including the subgroups of participants who exclusively used the research cigarettes they were assigned.

**Table 2:** Means at baseline (visit 4)* and end of randomized phase (visit 10) for each group and results of statistical comparisons based on linear regression models.

	Mean at Visit 4 (SD) Baseline Phase,	Mean at Visit 10 (SD) End of Randomization Phase	Difference Between Groups, Adjusted for Baseline [95% CI]	P-value for Between-Group Comparison at Visit 10, Adjusted for Baseline	P-value for Between-Group Comparison at Visit 10, Adjusted for Baseline and Significant Predictors#	P-value for Between-Group Comparison at Visit 10 Among Compliers Only, Adjusted for Baseline
**Biomarkers of Toxicant Exposure** *						
**Plasma cotinine, in ng/mL**						
RNC [n = 66]	244.4 (143.5)	82.8 (154.3)	-175.7 [-218.3, -133.1]	<0.0001	<0.0001	<0.0001
UNC [n = 72]	245.2 (126.5)	259.0 (151.3)				
**Exhaled CO, in ppm**						
RNC [n = 69]	30.8 (18.5)	21.4 (17.5)	-7.86 [-12.06, -3.66]	0.0003	0.08	0.0008
UNC [n = 73]	29.6 (15.5)	28.5 (15.8)				
**NNAL, in pmol/mg creatinine**						
RNC [n = 26]	1.0 (0.7)	0.71 (1.0)	-0.54 [-1.02, -0.06]	0.03	0.03	0.0004
UNC [n = 26]	1.1 (0.7)	1.3 (1.1)				
**GSSP: GSH ratio**						
RNC [n = 25]	0.14 (0.06)	0.18 (0.06)	-0.005 [-0.04, +0.03]	0.75	0.27	0.63
UNC [n = 24]	0.14 (0.06)	0.18 (0.05)				
**8-Isoprostanes, in ng/mg creatinine**						
RNC [n = 25]	4.3 (1.7)	4.0 (1.7)	-0.59 [-1.47, +0.28]	0.18	0.18	0.38
UNC [n = 25]	3.8 (1.6)	4.3 (2.1)				
**1-Hydroxypyrene, in ng/mg creatinine**						
RNC [n = 26]	0.3 (0.2)	0.2 (0.2)	-0.05 [-0.14, 0.05]	0.31	0.31	0.55
UNC [n = 26]	0.2 (0.1)	0.3 (0.2)				
**Cigarette Smoking Behaviors & Dependence**						
**Cigarettes per day**						
RNC [n = 69]	20.2 (11.0)	17.4 (16.1)	-4.53 [-7.43, -1.64]	0.002	0.002	0.02
UNC [n = 74]	21.8 (10.6)	23.7 (12.8)				
**FTCD total score**						
RNC [n = 68]	5.8 (2.5)	4.8 (2.9)	-1.18 [-1.67, -0.69]	<0.0001	<0.0001	<0.0001
UNC [n = 73]	6.0 (2.4)	6.2 (2.5)				
**PSCDI total score**						
RNC [n = 66]	12.6 (3.7)	10.6 (4.5)	-1.99 [-2.84, -1.14]	<0.0001	<0.0001	0.0001
UNC [n = 73]	13.0 (3.4)	13.0 (3.6)				
**HONC total score**						
RNC [n = 69]	7.7 (2.1)	7.0 (2.6)	-0.68 [-1.22, -0.14]	0.01	0.01	0.03
UNC [n = 74]	8.1 (2.0)	8.0 (2.1)				
**MNWS total score**						
RNC [n = 69]	8.9 (7.2)	8.1 (6.8)	-0.31 [-1.68, +1.05]	0.65	0.65	0.98
UNC [n = 74]	9.8 (6.0)	9.0 (5.9)				
**QSU total score**						
RNC [n = 69]	30.4 (15.5)	26.5 (15.5)	-3.77 [-7.79, +0.25]	0.07	0.63	0.008
UNC [n = 74]	33.4 (15.7)	32.1 (14.8)				
**Mental Health Indicators**						
**Kessler K6 total score**						
RNC [n = 69]	5.3 (5.5)	4.6 (4.7)	0.28 [-0.71, +1.28]	0.57	0.60	0.21
UNC [n = 73]	6.1 (4.7)	4.9 (4.6)				
**OASIS total score**						
RNC [n = 68]	4.5 (3.9)	4.5 (4.2)	0.60 [-0.41, +1.61]	0.24	0.24	0.34
UNC [n = 73]	5.2 (4.0)	4.4 (4.0)				
**QIDS total score**						
RNC [n = 69]	4.9 (4.6)	5.5 (4.3)	0.69 [-0.28, +1.65]	0.16	0.16	0.09
UNC [n = 72]	5.5 (3.7)	5.3 (3.9)				
**PSS total score**						
RNC [n = 69]	15.6 (7.8)	15.0 (7.6)	0.38 [-1.40, +2.16]	0.67	0.67	0.46
UNC [n = 74]	16.3 (8.2)	15.1 (7.8)				
**CES-D total score**						
RNC [n = 67]	17.1 (8.5)	17.0 (7.8)	1.05 [-0.95, +3.05]	0.30	0.30	0.36
UNC [n = 72]	17.9 (6.8)	16.4 (7.4)				
**Health Status Indicators**						
**Systolic BP, in mmHg**						
RNC [n = 69]	121.8 (12.6)	122.8 (13.9)	-0.78 [-4.48, +2.92]	0.68	0.58	0.55
UNC [n = 73]	121.6 (14.3)	123.4 (15.0)				
**Diastolic BP, in mmHg**						
RNC [n = 69]	78.7 (9.8)	79.0 (12.2)	-1.84 [-6.11, +2.42]	0.39	0.27	0.80
UNC [n = 73]	77.3 (9.7)	79.9 (16.3)				
**Heart rate, in bpm**						
RNC [n = 69]	81.2 (13.7)	76.2 (12.2)	-1.32 [-4.77, +2.14]	0.45	0.62	0.97
UNC [n = 73]	82.0 (12.5)	77.9 (12.3)				
**Weight, in pounds**						
RNC [n = 69]	198.4 (52.1)	198.1 (49.8)	0.68 [-4.08, +5.45]	0.78	0.94	1.00
UNC [n = 73]	193.4 (57.4)	192.7 (57.5)				
**Waist: Hip ratio**						
RNC [n = 69]	0.9 (0.1)	0.9 (0.1)	0.02 [-0.003, +0.04]	0.09	0.09	0.06
UNC [n = 73]	0.9 (0.1)	0.9 (0.1)				
**FEV1, in liters**						
RNC [n = 67]	2.8 (0.8)	2.8 (0.8)	-0.0006 [-0.07, +0.06]	1.00	0.99	0.69
UNC [n = 71]	2.7 (0.7)	2.7 (0.7)				
**CCQ total score**						
RNC [n = 69]	0.9 (0.8)	0.9 (0.9)	-0.05 [-0.23, +0.14]	0.64	0.61	0.32
UNC [n = 73]	1.0 (0.8)	1.0 (0.8)				

*Abbreviations: CO (carbon-monoxide), NNAL [4-(methylnitrosamino)-1-(3-pyridyl)-1-butanol], GSSP:GSH (Ratio of Glutathione to Oxidized Glutathione), FTCD (Fagerstom Test for Cigarette Dependence), HONC (Hooked on Nicotine Checklist), MNWS (Minnesota Nicotine Withdrawal Scale), QSU (Questionnaire on Smoking Urges-short form), OASIS (Overall Anxiety Severity and Impairment Scale), QIDS (Quick Inventory of Depressive Symptomatology), PSS (Perceived Stress Scale), CES-D (*Center for Epidemiological Studies-Depression Scale*). BP (Blood Pressure), FEV1 (Forced Expiratory Volume in 1 second), CCQ (Clinical COPD Questionnaire). Where the variable was not measured at visit 4 but was measured at an earlier visit (e.g. CES-D) the measurement at the earlier visit was used as the baseline. **#** The baseline covariates initially entered in the linear regression models were: Treatment group, site, cigarette flavor, BMI, height, weight, age, sex, race, education, time-to-first-cigarette, cigarettes per day, number of current MINI diagnoses (0, 1, 2 3+), plus the baseline measure of the outcome variable under analysis.

Table 2 summarizes the results of multivariable linear regression models. When comparing the two groups, adjusting only for the baseline variable, indicators of smoke exposure, plasma cotinine, exhaled CO and NNAL concentration, and measures of nicotine dependence, CPD, FTND, PSCDI, and the HONC were significantly lower at the end of the randomized phase in the RNC group as compared to the UNC group. All except one of these effects remained significant, both when controlling for other significant baseline predictors and when analyses only included those who were compliant with exclusive use of research cigarettes. The only exception was exhaled CO, which was not significant (p = 0.08) when controlling for significant baseline predictors. The QSU total score (10-item measure of strength of urges to smoke a cigarette) was only significantly lower in the RNC group when the analysis was restricted to those fully compliant with smoking the assigned research cigarettes. Assessments of psychiatric and nicotine withdrawal symptoms, CES-D, QIDS, OASIS, PSS and MNWS, showed no significant between group differences. There were also no significant effects of treatment group on health indicators or biomarkers of oxidative stress (glutathione, 8-isoprostanes). Linear mixed-effect models, incorporating data from all randomized participants and visits showed an identical pattern of results (full results shown in S3-S54 Figs in S3 File) with significant visit (time) by group interactions for cotinine, CPD, and CO but not for any of the mental health indicators. The minimal data set underlying these results (including means and SDs at each visit) can be found in S4 and S5 Files.

**Fig 2:**
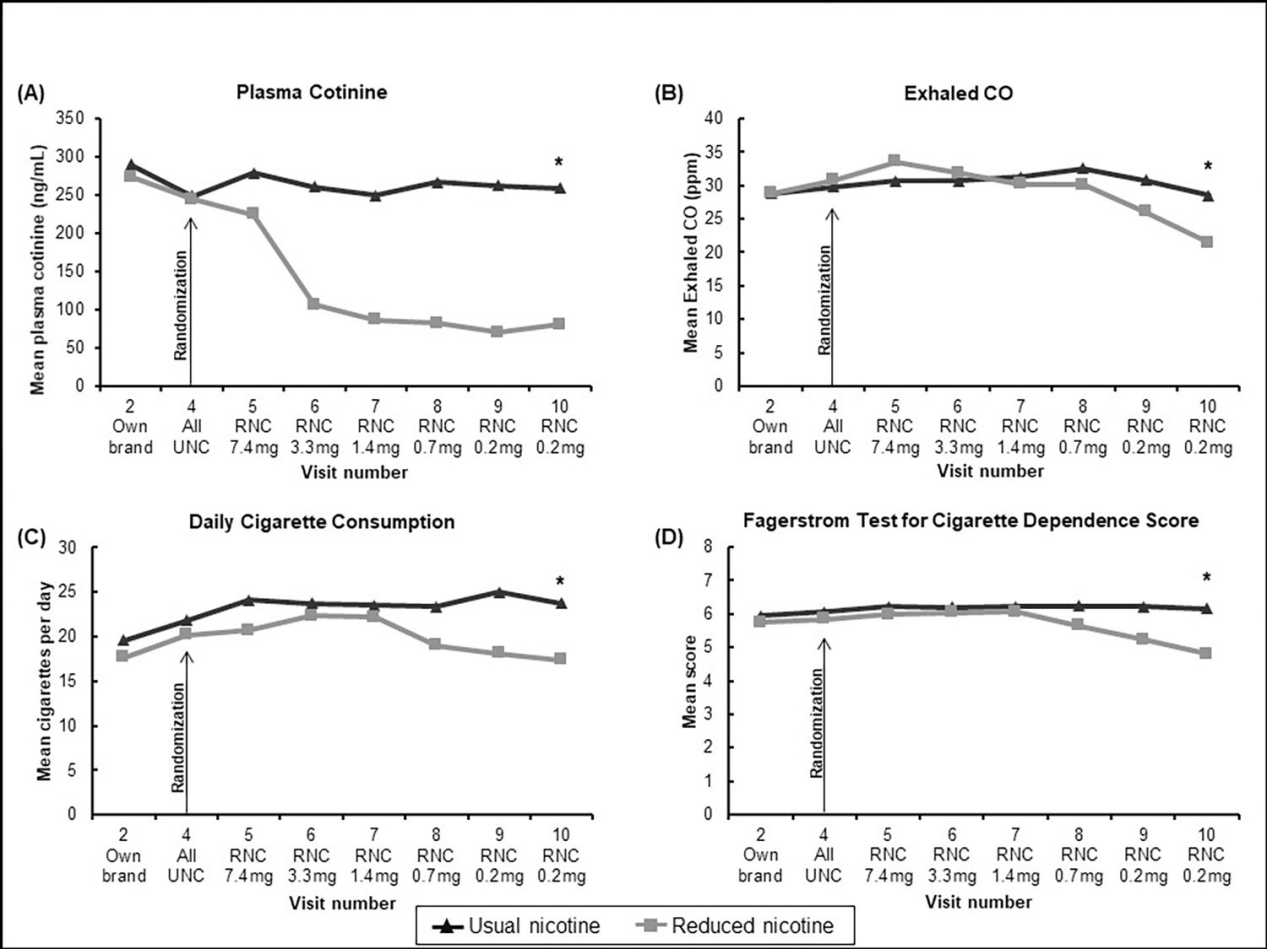
**2A-2D.** Changes in plasma cotinine, exhaled CO, daily cigarette consumption, and FTND nicotine dependence score among completers (n = 143) smoking either Usual Nicotine Content (n = 74) cigarettes or Reduced Nicotine Content (n = 69) cigarettes. * indicates statistically significant between group difference at Visit 10, controlling for Visit 4 baseline.

Fig 2 shows the primary outcome data for plasma cotinine, exhaled CO, daily cigarette consumption and the FTCD measure of nicotine dependence throughout the study.

**Fig 3:**
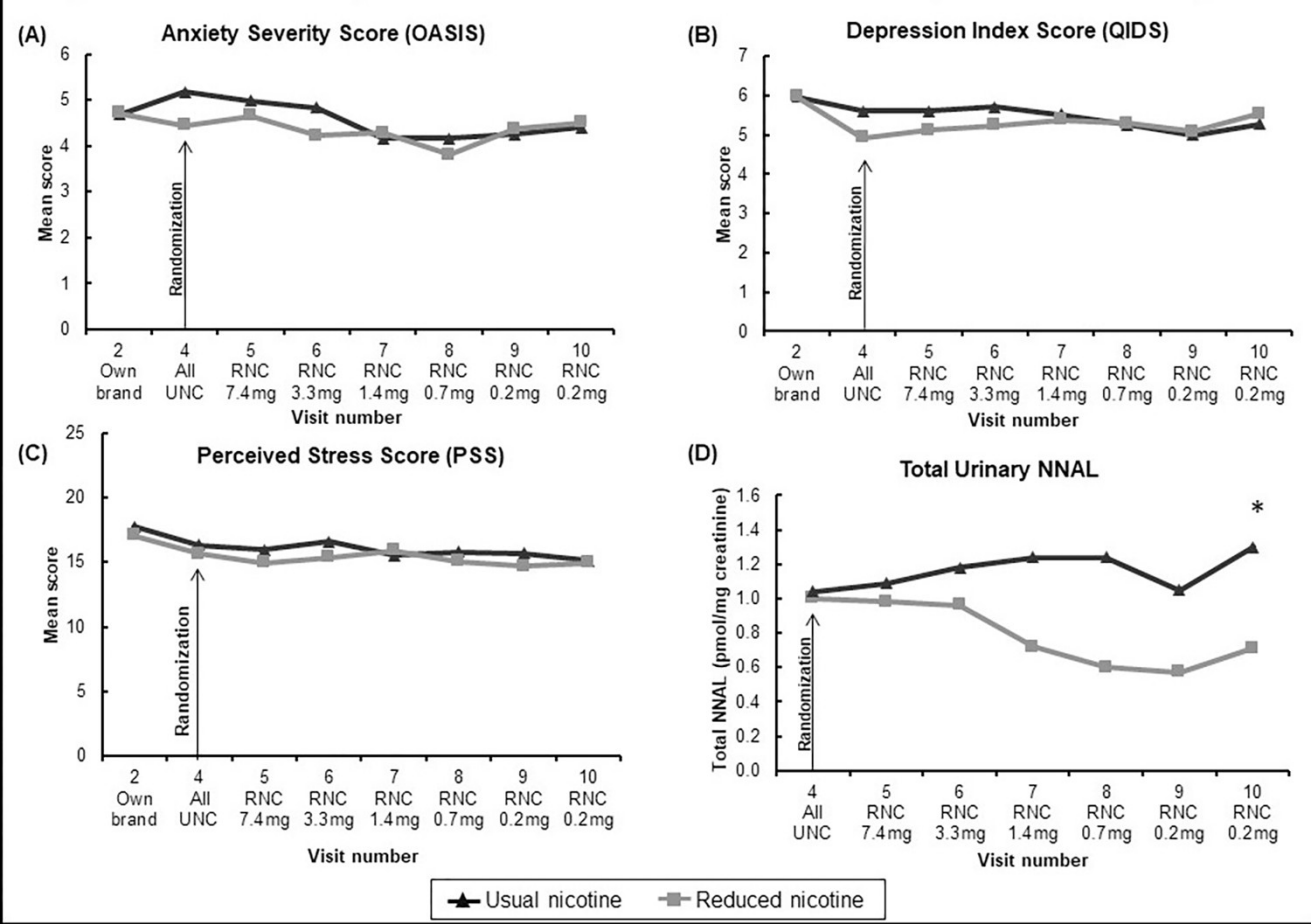
**3A-3D.** Changes in OASIS, QIDS, PSS, and NNAL^#^ among completers (n = 143) smoking either Usual Nicotine Content (n = 74) cigarettes or Reduced Nicotine Content (n = 69) cigarettes. * indicates statistically significant between group difference at Visit 10, controlling for Visit 4 baseline. # urine NNAL measured in randomly selected subgroup (n = 26 on RNCs and n = 25 on UNCs, evenly stratified by site and by study group), who provided urine samples at randomization (v4) and at the end of the randomized phase of the trial (v10).

Fig 3 shows the outcome data for mental health measures (OASIS, QIDS, PSS) and the carcinogen exposure biomarker NNAL throughout the study. The other mental health and general health indicators showed similar patterns with no significant differences between groups.

### Protocol adherence

Adherence with the study protocol to smoke only the assigned research cigarettes during the trial was imperfect for both groups, with 62/74 (83.8%) participants in the UNC and 41/69 (59.4%) in the RNC group meeting self-report and biochemical criteria for strict adherence, defined as non-use of non-research cigarettes [39]. Reported use of other non-cigarette nicotine products was rare, occurring on 3 occasions in the UNC group and 4 occasions in the RNC group during the randomized phase (visits 5 through 10). The overall pattern of results comparing the subgroups in each arm with strict adherence was very similar to the results reported above for study completers. The S3 File provides figures for each outcome showing the pattern of change in each group among (a) all completers to visit 10 (b) all completers who were compliant with their assigned research cigarettes at visit 10 (compliers) and (c) for main outcome measures [per protocol] the pattern of results for all participants attending each visit (n which varied by visit).

### Adverse events

A total of 144/188 participants (76.6%) reported at least one adverse event (AE) during the randomized phase of the trial, with very similar frequencies in the two groups: 75.5% in the RNC group and 77.7% of the UNC group. Two-thirds (215/327) of the AEs were considered “mild”. Thirteen serious adverse events (SAEs) occurred in 12 participants during the randomized phase; 4 among participants randomized to RNC cigarettes and 9 among those randomized to UNC cigarettes. Three of these SAEs were psychiatric, 1 in the RNC group and two in the UNC group. Eight of these participants were withdrawn from the trial (3 on RNCs, 5 on UNCs) due to their SAE. Details of AEs are in the S4-S10 Tables in S3 File.

### Increases in use of psychiatric medications

As shown in Table 1, more than half of each group was using a psychiatric medication at enrollment. Ten participants (10.6%) in the UNC group and 12 (12.8%) in the RNC group increased their dose or started a new psychiatric medication during the trial. Of participants who were not taking a psychiatric medication at randomization, 4 participants in the UNC and 5 participants in the RNC group started taking a psychiatric medication during the randomized phase.

### Treatment choice and smoking cessation

143 participants attended the last randomized phase visit and entered the treatment choice phase of the trial. 33/69 (47.8%) of those on RNCs and 25/74 (33.8%) on UNC cigarettes chose to try to quit smoking; 25/69 (36.2%) of those on RNCs and 46/74 (62.2%) on UNCs chose to continue smoking study cigarettes, while 11/69 (15.9%) on RNC and 3/74 (4.1%) on UNC chose to return to smoking their own brand cigarettes. The association between the treatment choice and study arms was significant, (chi-squared test, p = 0.003**).**

**Fig 4:**
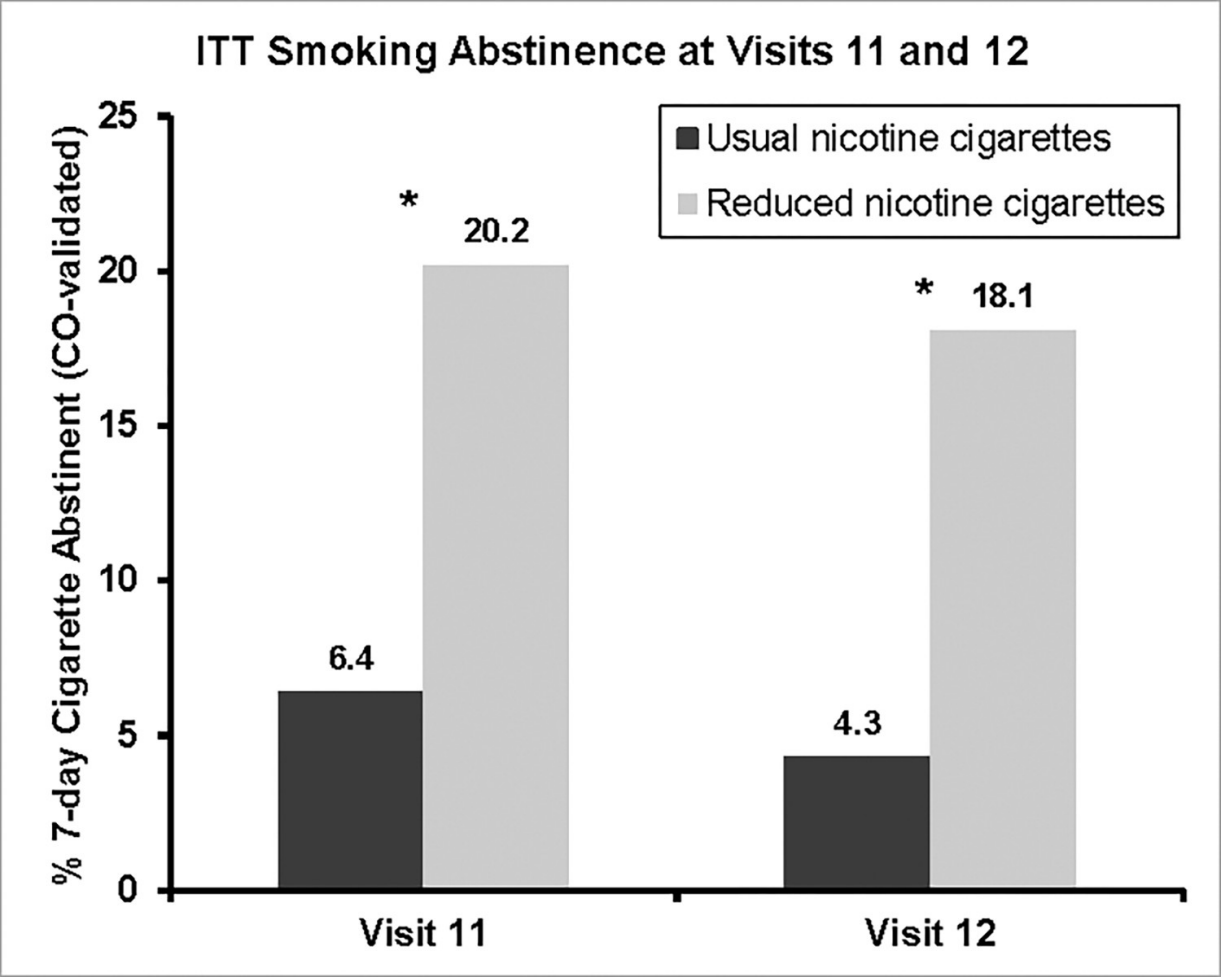
The proportion of participants initially randomized to reduced nicotine content or usual nicotine content cigarettes who had quit smoking cigarettes at visit 11, and at visit 12. *p<0.01 for comparison between proportion randomized to RNC and UNC group who reported no cigarettes smoked in the previous week with exhaled CO<10ppm.

At the end of the treatment choice phase (visit 12), 17/94 (18.1%) of the RNC group and 4/94 (4.3%) in the UNC group met study criteria for abstinence, defined as self-report of no tobacco use in the previous 7 days and exhaled CO <10ppm, assuming dropouts to be smokers, Fisher’s exact test, p = 0.004. The mean CO of those abstinent at visit 12 was <4ppm for both groups and all but two (one of each group) were also abstinent 8 weeks earlier (visit 11). The intent-to-treat abstinence rates at visits 11 and 12 are shown in Fig 4.

## Discussion

This study found that when smokers with affective disorders switch to cigarettes with gradually reduced nicotine content, they have progressively lower plasma cotinine, and once they are smoking cigarettes with very low nicotine content they smoke fewer cigarettes per day, have a lower exhaled CO and report being less addicted to their cigarettes than smokers randomly assigned to continue smoking usual nicotine cigarettes. We found no evidence that using reduced nicotine cigarettes, versus UNCs, was associated with worsening general health or mental health problems or adverse events. When offered a choice to quit smoking, more of those randomized to RNC cigarettes succeeded in quitting smoking over 12 weeks, despite the fact that only slightly more of the RNC group (n = 33) than the UNC group (n = 25) chose to try to quit. To our knowledge, this is the first randomized trial of reduced nicotine cigarettes in smokers with affective disorders to find that randomization to RNC cigarettes was associated with significantly increased rates of biochemically-validated smoking cessation. The higher biochemically validated quit rate in the RNC group is consistent with the lower measured dependence in that group towards the end of the randomized phase of the trial.

The lack of significant effects on some biomarkers of toxicant exposure (glutathione, 8-isoprostanes and 1-hydroxypyrene) may reflect the presence of oxidants and combustion products in the smoke from the RNC cigarettes but may also be a result of the small number of completer samples (25–26 per group) causing limited statistical power. A much larger trial of very low nicotine cigarettes [12] also found very few effects on biomarkers of inflammation, oxidative stress and hematological parameters. It did, however, find significantly lower quantities of urine phenanthrene tetraol (PheT), in smokers randomized to either immediate or gradual nicotine reduction in their cigarettes, as compared with a normal nicotine cigarette control group at 20 week follow-up. Phet, like 1-hydroxypyrene is an indicator of polycyclic aromatic hydrocarbon exposure. They found a 10–15% reduction in Phet exposure, which may not have been detectable in the current study with far fewer samples.

The eligibility criteria, participant payments and provision of free research cigarettes may impact the generalizability of the study findings. Other limitations of the study include the facts that 23.9% of the participants did not complete the randomized phase and imperfect adherence to the protocol for exclusive use of research cigarettes in those who did complete the trial. However, the rate of dropout was similar in the two groups and was anticipated in the trial protocol, which aimed to conduct the main analyses on approximately 70 completers in each group. The pattern of results was virtually identical for per protocol analyses as for analyses of all completers. While the trial included smokers with a lifetime history of mood and/or anxiety disorders and did not require a current mood or anxiety disorder for enrollment, and those who were recently suicidal or had recently received inpatient psychiatric treatment were excluded, almost a third of the sample had a history of attempted suicide, suggesting that the sample was at high risk of worsening mental health. However, this study did not find that switching to RNC cigarettes worsens mental health.

The present study is consistent with others [10, 13, 14] in being broadly reassuring about the effects of switching to very low nicotine cigarettes on mental health outcomes in those with mental disorders, and adds the findings of reduced toxicant exposure and increased probability of successful smoking cessation when treatment is offered. A recent trial [12] that excluded smokers with serious psychiatric illness demonstrated that abrupt nicotine reduction in cigarettes is feasible. Future research should examine the effects of abrupt nicotine reduction in cigarettes on smokers with psychiatric conditions, and also assess the effects of availability of other non-combusted nicotine sources (e.g. electronic cigarettes or oral nicotine products) on the effects of abrupt nicotine reduction in cigarettes.

The governments of the United States and New Zealand have both recently proposed a nicotine reduction policy for cigarettes and most other smoked tobacco products [2–4]. The results of this study suggests that such a policy will likely result in reduced nicotine absorption from cigarettes without worsening the mental health of smokers with mood or anxiety disorders. It also suggests that, so long as other “cleaner” sources of nicotine are available for smokers to transition to, and brief support is available, a nicotine reduction policy will likely result in more smokers with mood and anxiety disorders quitting smoking.

## Conclusion

Lowering the permissible nicotine content in cigarettes to very low levels over 15 weeks reduces toxicant exposure and increases smoking cessation without worsening mental health among smokers with mood or anxiety disorders.

## Supporting information

S1 Checklist (DOC)

S1 File (PDF)

S2 File (PDF)

S3 File (PDF)

S4 File (PDF)

S5 File (PDF)

## Abbreviations

NNAL: 4-(methylnitrosamino)-1-(3-pyridyl)-1-butanol
NNK: 4-(methylnitrosamino)-1-(3-pyridyl)-1-butanone
CO: carbon monoxide
DSM-5: Diagnostic and Statistical Manual of Mental Disorders, 5th Edition
