# Supplementary material for: The effects of reduced nicotine content cigarettes on biomarkers of nicotine and toxicant exposure, smoking behavior and psychiatric symptoms in smokers with mood or anxiety disorders: A double-blind randomized trial
Source: PLoS One. 2022 Nov 2;17(11):e0275522. doi: 10.1371/journal.pone.0275522 (PMC9629593; doi:10.1371/journal.pone.0275522)
Supplement: S5 File — (PDF) [file pone.0275522.s006.pdf]

The effects of reduced nicotine content cigarettes on biomarkers of nicotine and toxicant exposure, smoking behavior and psychiatric symptoms in smokers with mood or anxiety disorders: a double-blind randomized trial (Foulds et al). - Continuous outcomes variables between treatment groups across all visits (for only participants who completed the randomized phase through visit 10)

Penn State TCORS biostatistics core

Sept. 1, 2022

Contents

Consumption: Total CPD (Cig Log - Full 6 Day FB). 3
Consumption: Study CPD (Cig Log - Full 6 Day FB). 4
Biomarker: Cotinine (Plasma - < LOD = 3). 5
Biomarker: CO. 6
Nicotine Dependence: FTND. 7
Nicotine Dependence: Fagerstrom Test for Nicotine Dependence (w/o CPD). 8
Nicotine Dependence: PSCDI. 9
Nicotine Dependence: Penn State Cigarette Dependence Index (w/o CPD). 10
Nicotine Withdrawal: MNWS. 11
Psychiatry: QIDS. 12
Psychiatry: OASIS. 13
Psychiatry: Kessler K6. 14
Psychiatry: Perceived Stress. 15
Psychiatry: CES-D. 16
Adverse Health Effects: CCQ - COPD Total. 17
Adverse Health Effects: Systolic Blood Pressure. 18
Adverse Health Effects: Diastolic Blood Pressure. 19
Pulse. 20
Weight. 21
Questionnaire on Smoking Urges. 22
Adverse Health Effects: FEV1 Liters. 23
Biomarker: GSSP/GSH Ratio. 24
Biomarker: (Standardized) Total Urine 8-Isoprostanes (ng/mg creatinine). 25
Biomarker: (Standardized) Total Urine NNAL (pmol/mg creatinine). 26
Alcohol consumption. 27

In this report we calculated means (and standard deviations) for several main continuous outcome variables at each visit for the Reduced Nicotine Content Group (RNC) and Usual Nicotine Content group (UNC).

Note that the results are based on ALL participants who completed the randomized phase (through visit 10).

The data was downloaded from REDCap. The data has a total of **143** participants who completed the study: 69 in RNC, and 74 in UNC.

The plots show the mean and standard error of mean (SEM) values of the specific quantitative variable at each visit.

Consumption: Total CPD (Cig Log - Full 6 Day FB).

Visit	N for RNC	mean (SD) of Total CPD for RNC	N for UNC	mean (SD) of Total CPD for UNC
2	69	17.7 (10.51)	74	19.61 (10.21)
3	69	19.54 (10.05)	74	21.93 (11.12)
4	69	20.18 (10.96)	74	21.82 (10.62)
5	69	20.67 (10.77)	74	24.11 (12.33)
6	69	22.35 (13.14)	74	23.76 (12.45)
7	68	22.16 (14.88)	74	23.49 (11.68)
8	68	18.98 (13.48)	74	23.4 (12.61)
9	69	18.1 (15.55)	73	25.02 (13.17)
10	69	17.41 (16.08)	74	23.7 (12.82)

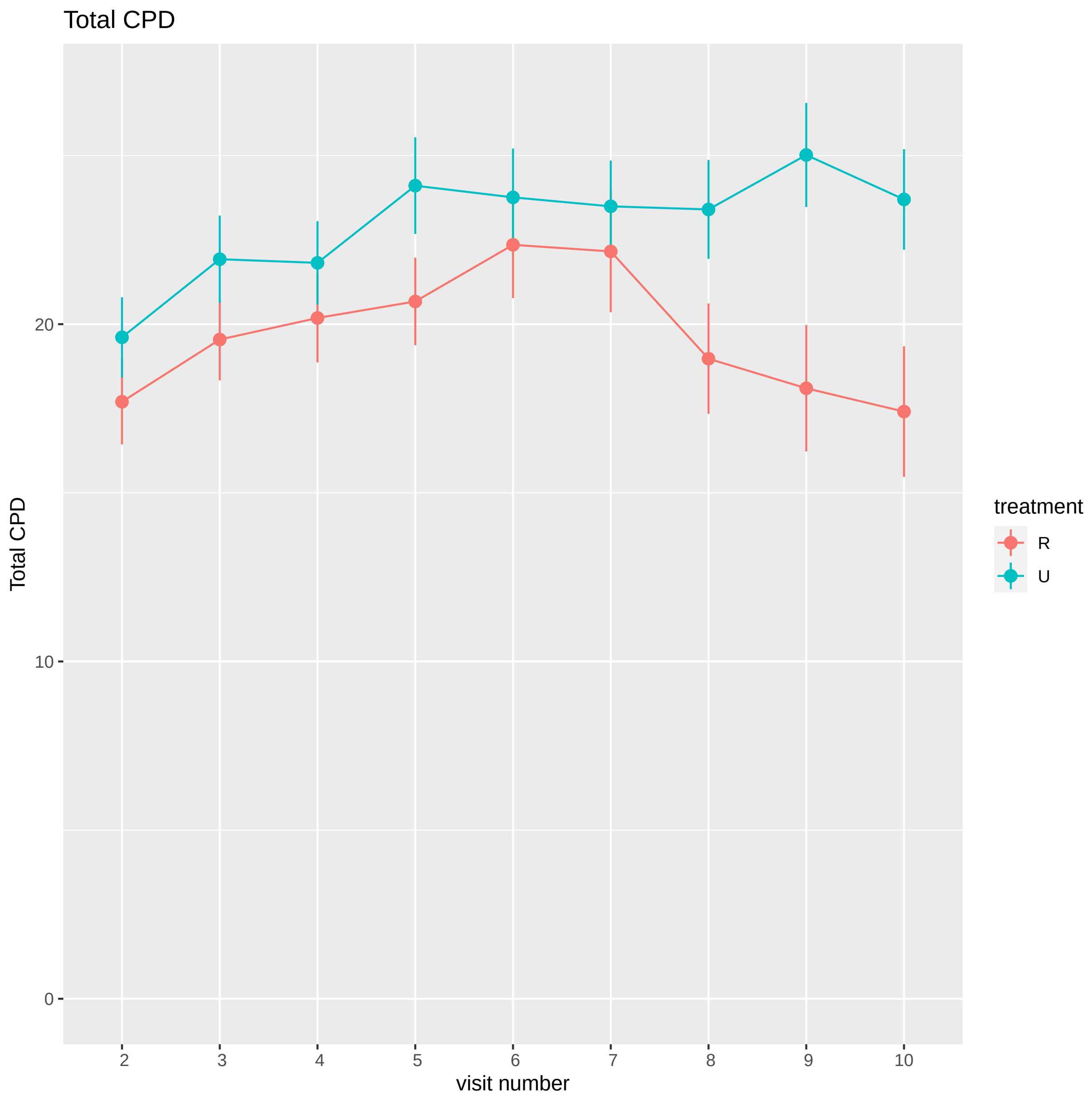

Consumption: Study CPD (Cig Log - Full 6 Day FB).

Visit	N for RNC	mean (SD) of Study CPD for RNC	N for UNC	mean (SD) of Study CPD for UNC
2	69	17.7 (10.51)	74	19.61 (10.21)
3	69	19.54 (10.06)	74	21.9 (11.12)
4	69	20.17 (10.97)	74	21.74 (10.62)
5	69	20.64 (10.78)	74	24.07 (12.35)
6	69	22.13 (13.25)	74	23.76 (12.45)
7	68	21.63 (14.94)	74	23.19 (12.03)
8	68	18.39 (13.8)	74	23.02 (12.87)
9	69	17.76 (15.65)	73	24.35 (13.77)
10	69	16.65 (16.25)	74	22.57 (13.4)

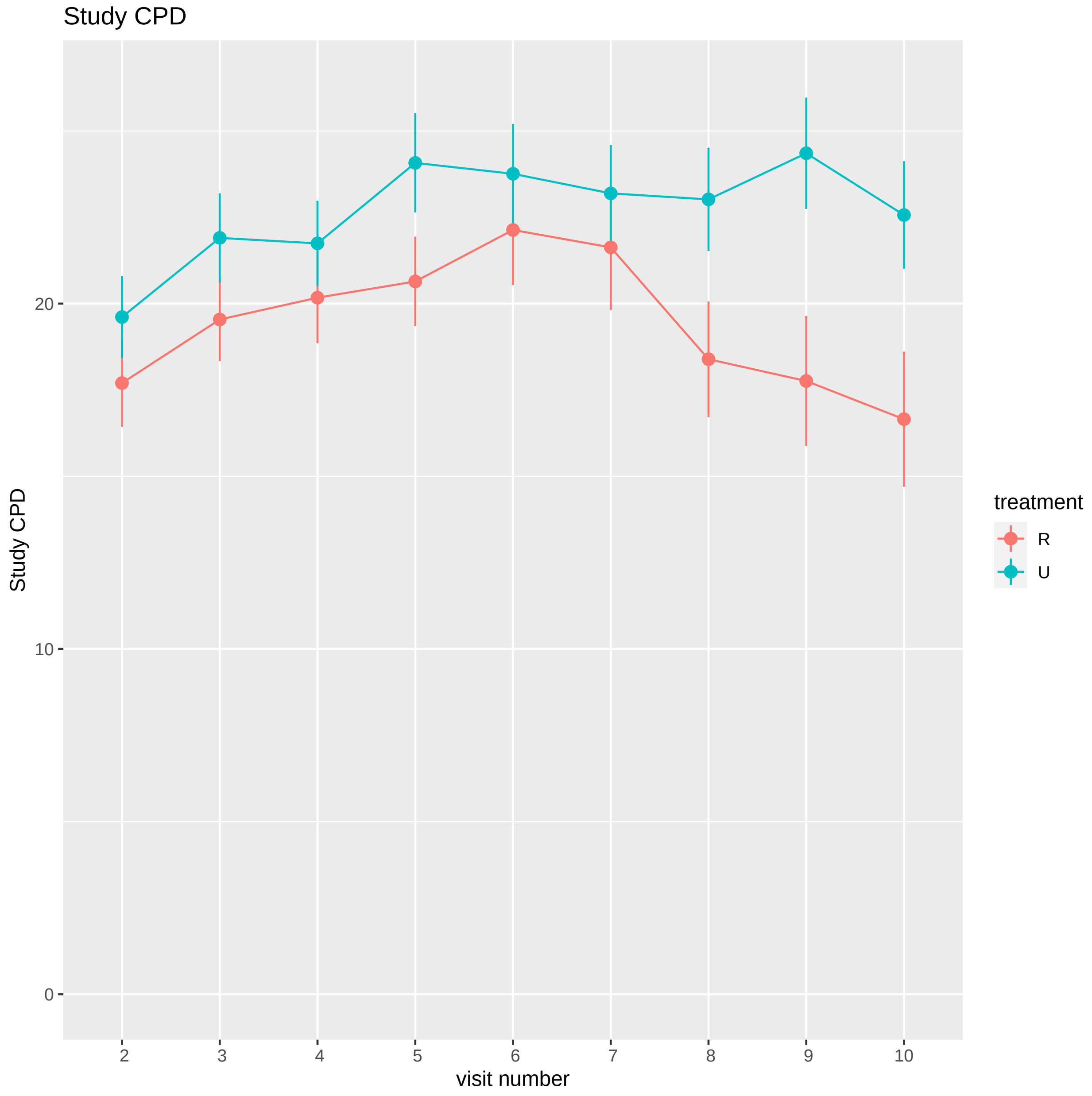

Biomarker: Cotinine (Plasma - < LOD = 3).

Visit	N for RNC	mean (SD) of Plasma Cotinine for RNC	N for UNC	mean (SD) of Plasma Cotinine for UNC
2	68	273.84 (144.58)	74	290.3 (154.1)
4	66	244.39 (143.51)	74	248.73 (126.69)
5	67	224.41 (141.21)	71	278.81 (157.47)
6	67	106.14 (80.97)	71	260.96 (148.38)
7	68	86.79 (105.7)	72	249.55 (144.27)
8	68	82.51 (151)	74	267.23 (147.04)
9	66	69.85 (131.97)	72	262.85 (152.92)
10	68	80.42 (152.54)	72	259.01 (151.3)

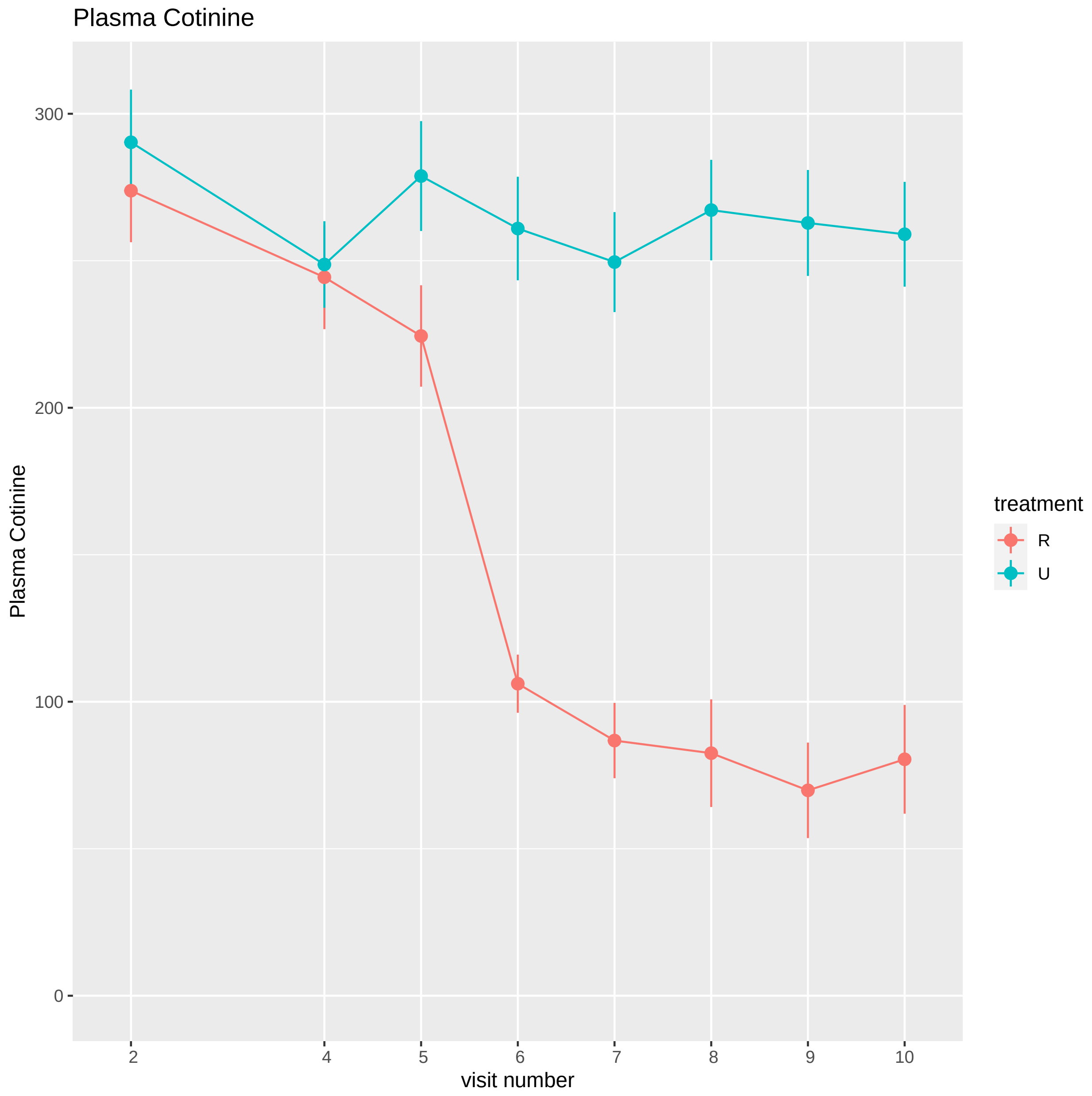

Biomarker: CO.

Visit	N for RNC	mean (SD) of Exhaled CO for RNC	N for UNC	mean (SD) of Exhaled CO for UNC
2	69	28.81 (18.15)	74	28.78 (16.64)
3	69	29.9 (18.17)	74	31.23 (17.45)
4	69	30.8 (18.48)	74	29.8 (15.55)
5	69	33.58 (20.58)	74	30.68 (16.49)
6	69	31.87 (18.88)	74	30.73 (17.64)
7	68	30.25 (18.28)	74	31.31 (16.97)
8	69	30.07 (20.27)	74	32.61 (18.75)
9	69	26.06 (18.78)	73	30.84 (16.81)
10	69	21.43 (17.49)	73	28.49 (15.82)

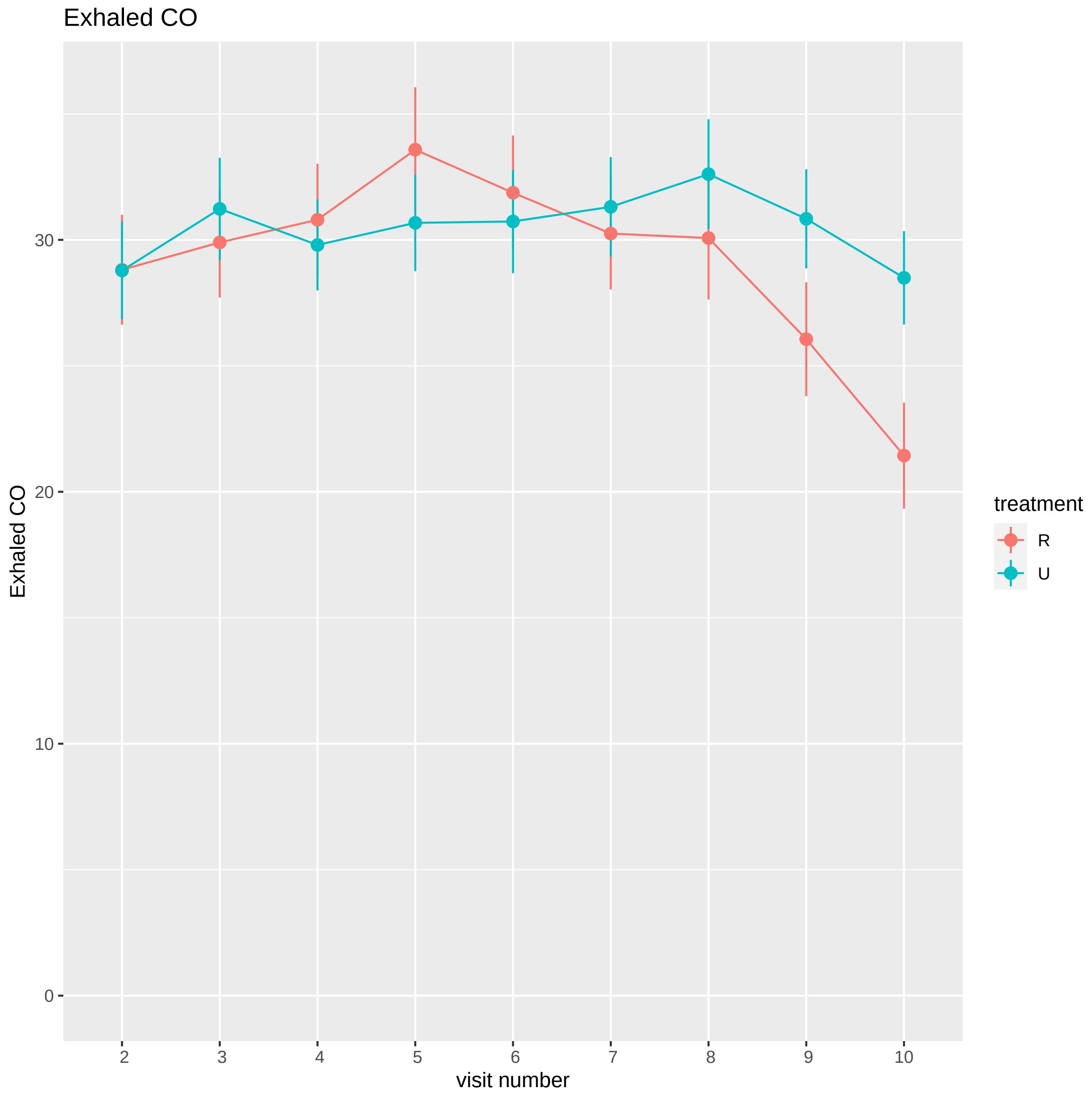

Nicotine Dependence: FTND.

Visit	N for RNC	mean (SD) of FTND for RNC	N for UNC	mean (SD) of FTND for UNC
2	69	5.75 (2.44)	74	5.95 (2.26)
3	69	5.83 (2.45)	74	6 (2.33)
4	69	5.84 (2.48)	74	6.07 (2.41)
5	69	5.99 (2.55)	74	6.22 (2.51)
6	69	6.04 (2.51)	74	6.19 (2.41)
7	68	6.06 (2.69)	72	6.22 (2.5)
8	69	5.64 (2.78)	74	6.24 (2.51)
9	68	5.24 (2.8)	72	6.22 (2.59)
10	68	4.79 (2.92)	73	6.16 (2.53)

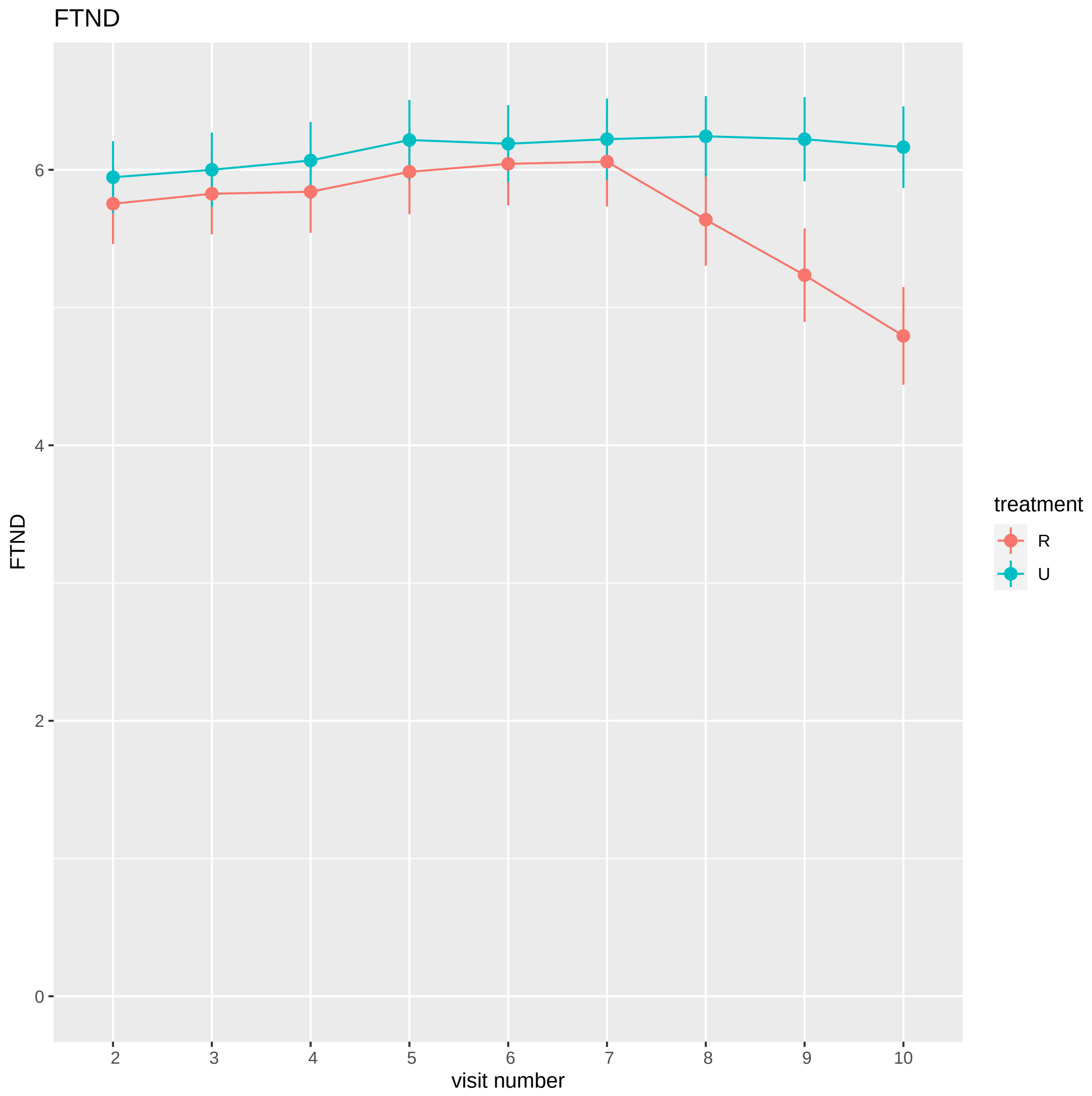

Nicotine Dependence: Fagerstrom Test for Nicotine Dependence (w/o CPD).

Visit	N for RNC	mean (SD) of FTND without CPD for RNC	N for UNC	mean (SD) of FTND without CPD for UNC
2	69	4.57 (1.91)	74	4.54 (1.75)
3	69	4.61 (1.9)	74	4.57 (1.81)
4	69	4.57 (1.94)	74	4.57 (1.89)
5	69	4.59 (2.05)	74	4.61 (1.91)
6	69	4.58 (1.94)	74	4.59 (1.88)
7	68	4.51 (2.06)	72	4.58 (1.98)
8	69	4.26 (2.14)	74	4.58 (1.94)
9	68	4.01 (2.17)	72	4.49 (1.99)
10	68	3.74 (2.27)	73	4.51 (1.92)

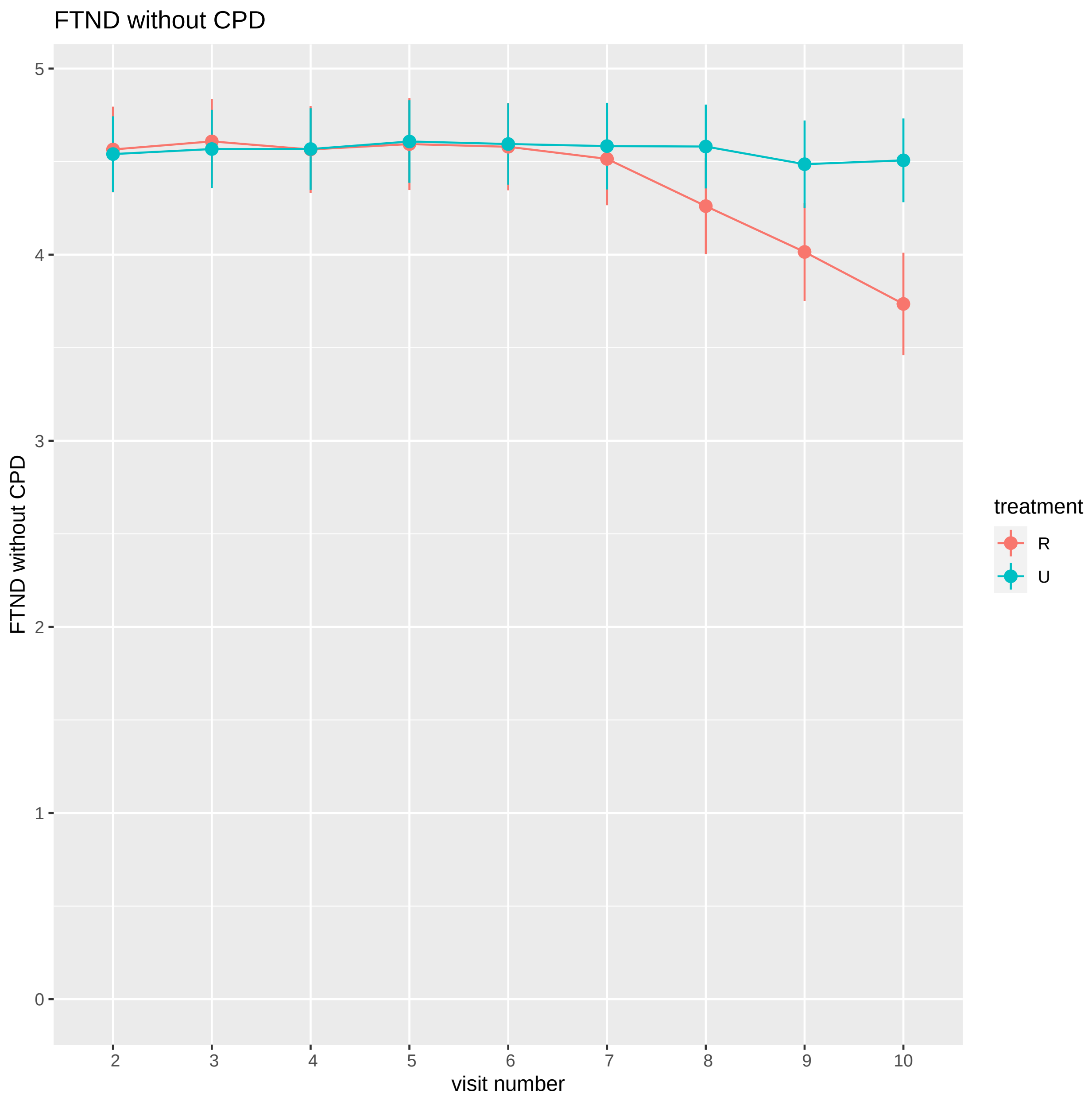

Nicotine Dependence: PSCDI.

Visit	N for RNC	mean (SD) of PSUCDI for RNC	N for UNC	mean (SD) of PSUCDI for UNC
2	68	12.59 (3.54)	74	13.38 (3.36)
3	69	12.72 (3.65)	74	13.3 (3.28)
4	69	12.49 (3.68)	74	13.12 (3.44)
5	69	12.8 (3.79)	73	13.41 (3.6)
6	69	12.93 (3.65)	74	12.97 (3.66)
7	69	12.46 (4.19)	72	13.35 (3.7)
8	69	12 (4.28)	73	13.05 (3.49)
9	67	11.37 (4.4)	72	13.25 (3.74)
10	66	10.61 (4.5)	73	13.03 (3.59)

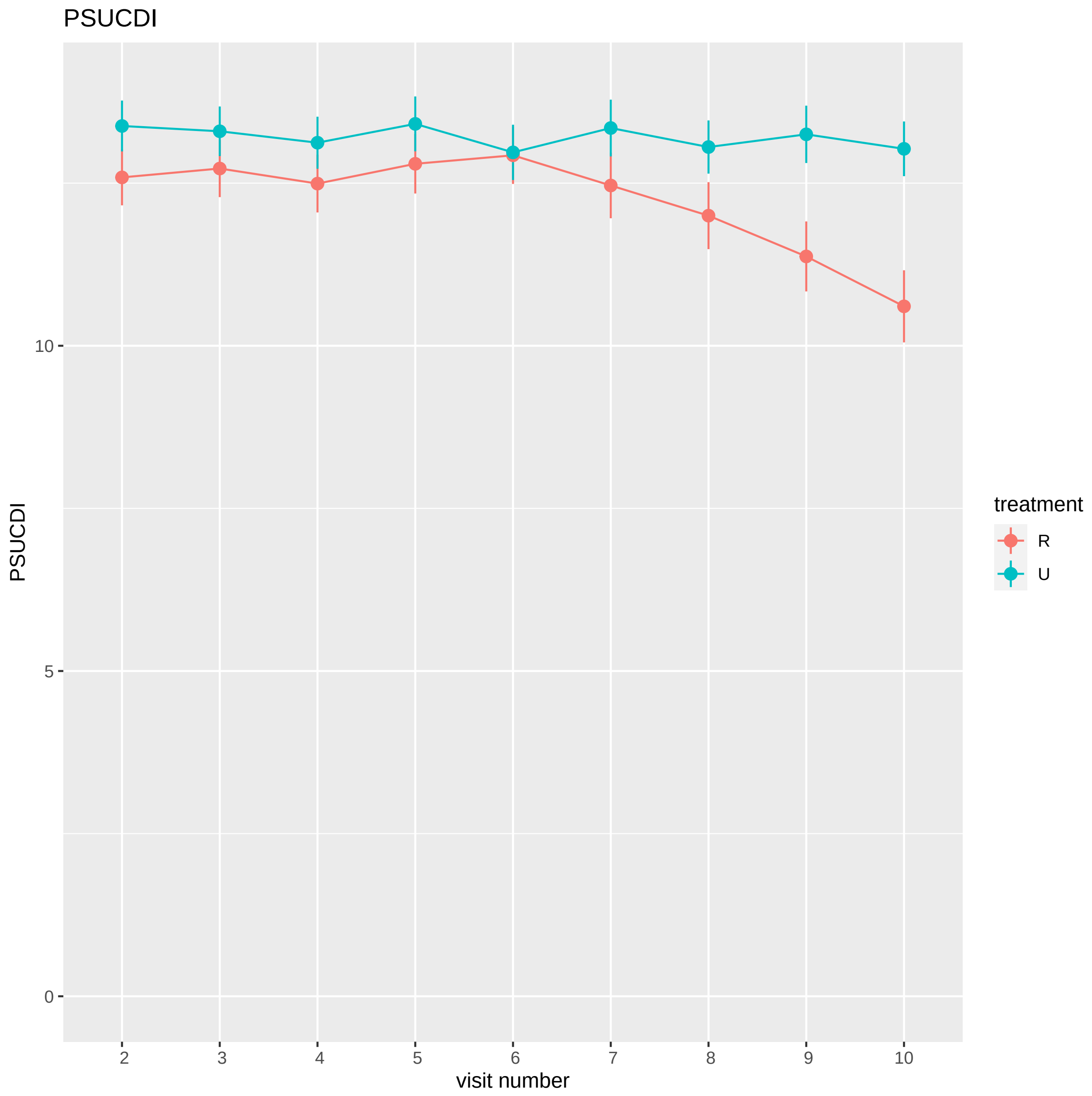

Nicotine Dependence: Penn State Cigarette Dependence Index (w/o CPD).

Visit	N for RNC	mean (SD) of PSUCDI without CPD for RNC	N for UNC	mean (SD) of PSUCDI without CPD for UNC
2	68	9.5 (2.8)	74	10 (2.77)
3	69	9.65 (2.8)	74	9.88 (2.59)
4	69	9.41 (2.91)	74	9.69 (2.83)
5	69	9.51 (3.13)	73	9.81 (2.87)
6	69	9.55 (2.89)	74	9.41 (2.94)
7	69	9.1 (3.3)	72	9.72 (3.04)
8	69	8.75 (3.26)	73	9.42 (2.78)
9	67	8.39 (3.48)	72	9.62 (2.99)
10	66	7.92 (3.36)	73	9.42 (2.82)

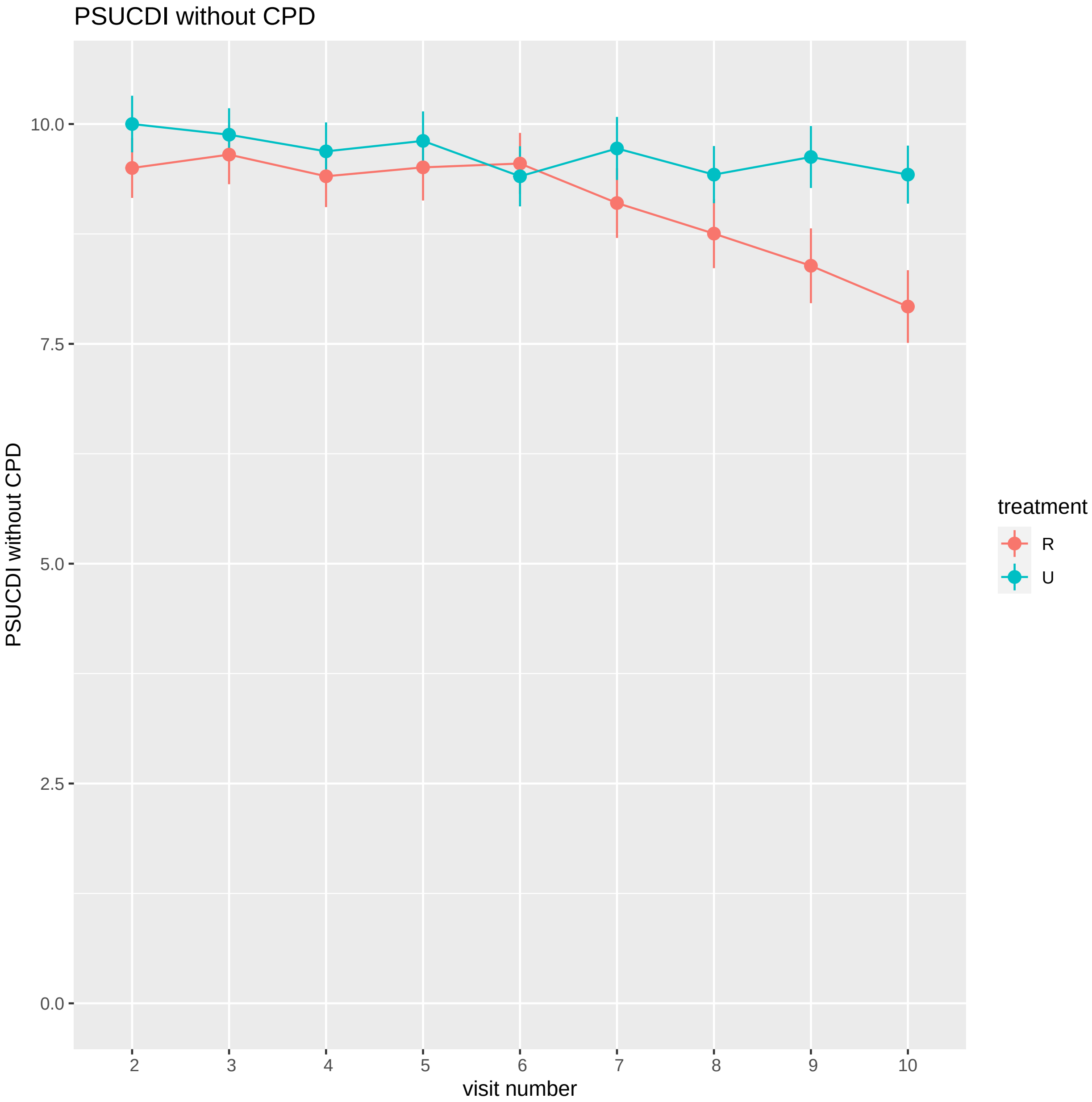

Nicotine Withdrawal: MNWS.

Visit	N for RNC	mean (SD) of MNWS for RNC	N for UNC	mean (SD) of MNWS for UNC
2	68	9.54 (6.93)	74	9.82 (5.72)
3	69	11.09 (7.44)	74	10.73 (6.25)
4	69	8.94 (7.15)	74	9.84 (6.02)
5	69	8.48 (6.15)	74	8.97 (6.28)
6	69	9.72 (7.24)	74	9.14 (5.82)
7	69	8.28 (7.33)	74	9.15 (5.83)
8	69	8.33 (7.13)	74	8.18 (5.36)
9	69	8.62 (7.5)	73	8.56 (5.45)
10	69	8.07 (6.77)	74	9.04 (5.91)

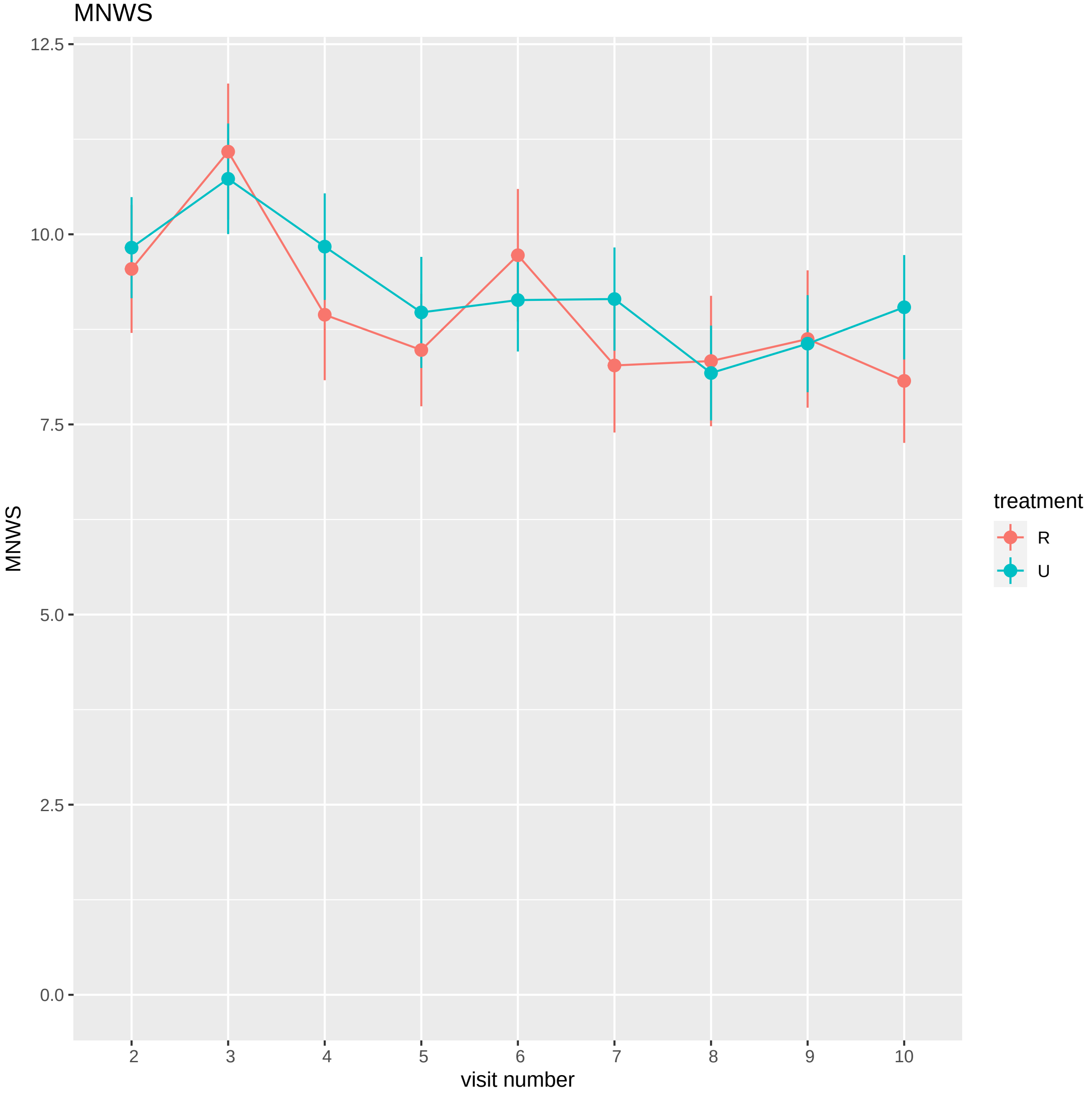

Psychiatry: QIDS.

Visit	N for RNC	mean (SD) of Depression: QIDS for RNC	N for UNC	mean (SD) of Depression: QIDS for UNC
2	69	5.96 (4.44)	72	5.96 (3.8)
4	69	4.93 (4.61)	73	5.6 (3.67)
5	69	5.12 (4.17)	72	5.61 (3.7)
6	69	5.23 (4.57)	72	5.71 (3.76)
7	68	5.37 (4.29)	73	5.51 (3.5)
8	69	5.29 (4.41)	73	5.26 (3.61)
9	68	5.09 (4.56)	71	4.99 (3.16)
10	69	5.54 (4.3)	72	5.28 (3.86)

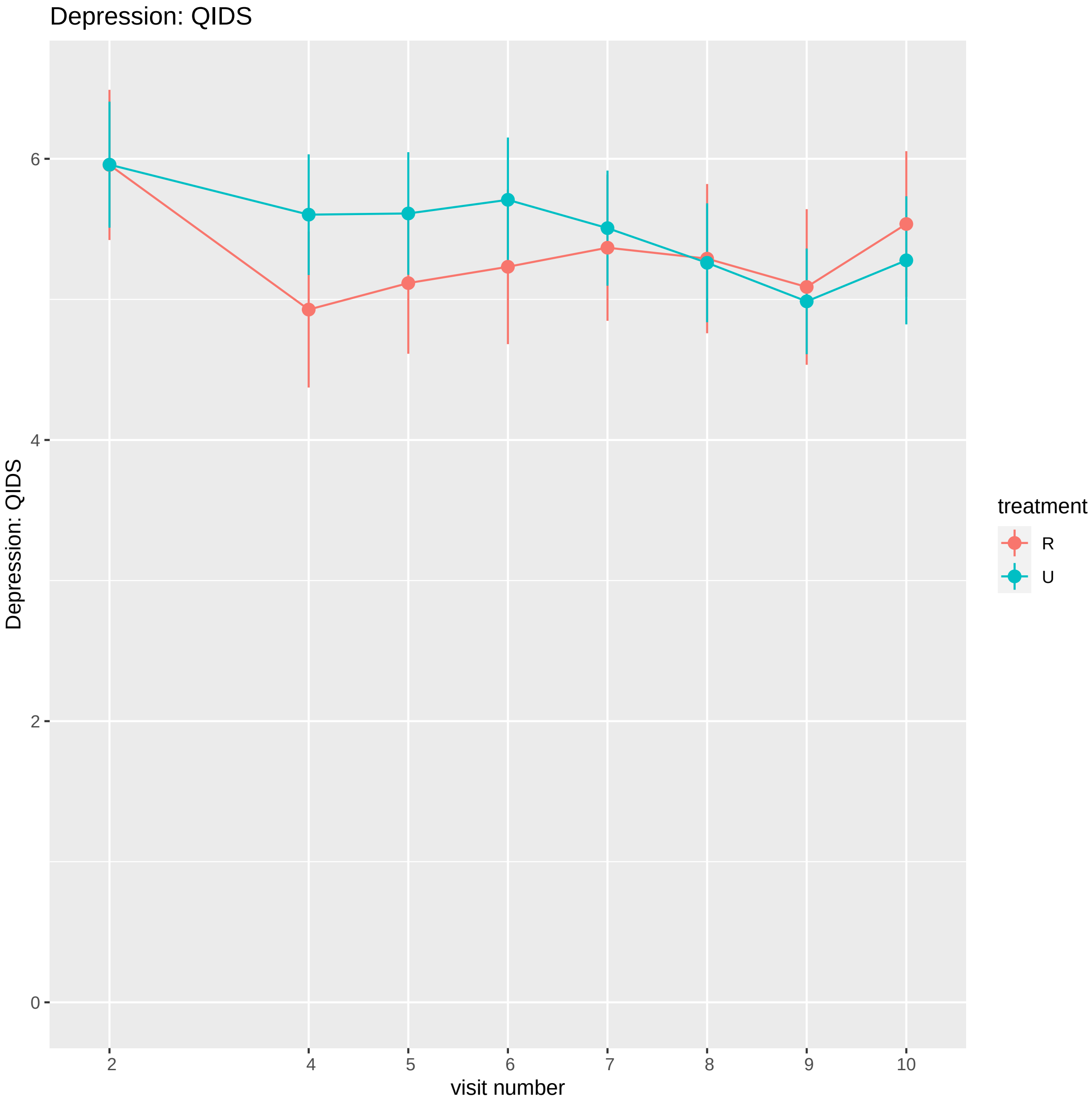

Psychiatry: OASIS.

Visit	N for RNC	mean (SD) of Anxiety: OASIS for RNC	N for UNC	mean (SD) of Anxiety: OASIS for UNC
2	69	4.71 (4.15)	73	4.68 (3.96)
4	69	4.45 (3.86)	74	5.18 (3.95)
5	69	4.65 (4.44)	74	4.99 (4.18)
6	69	4.23 (3.79)	74	4.84 (4.25)
7	69	4.28 (3.86)	73	4.16 (3.95)
8	69	3.81 (3.64)	74	4.16 (3.41)
9	68	4.37 (3.96)	72	4.25 (3.41)
10	68	4.51 (4.2)	73	4.4 (4.02)

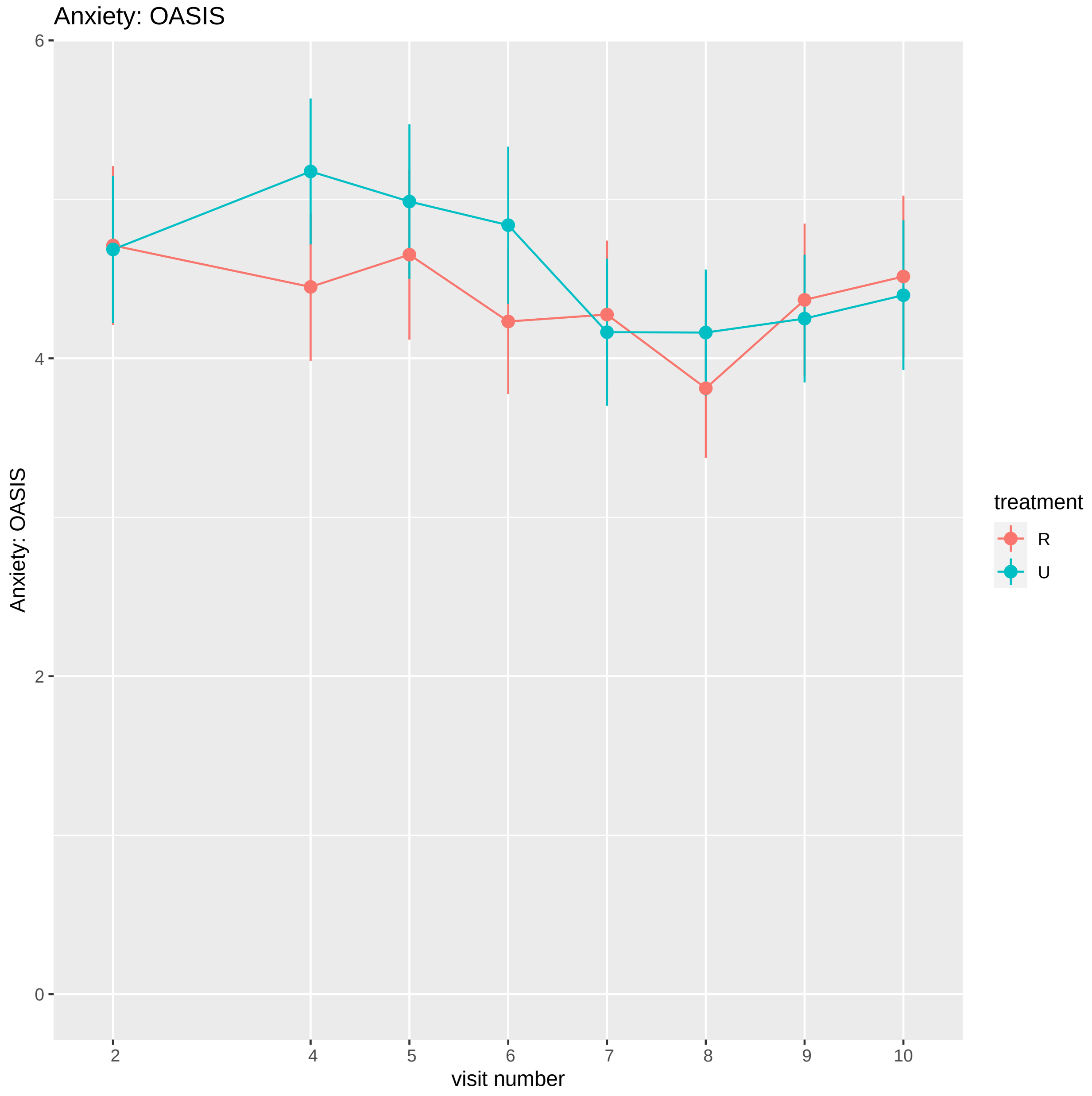

Psychiatry: Kessler K6.

Visit	N for RNC	mean (SD) of Kessler K6 for RNC	N for UNC	mean (SD) of Kessler K6 for UNC
2	68	5.43 (5.06)	74	6.23 (4.71)
4	69	5.29 (5.53)	73	6.08 (4.67)
5	69	4.57 (4.71)	74	5.22 (4.15)
6	69	4.99 (4.99)	74	5.76 (4.89)
7	69	4.72 (4.77)	74	5.2 (4.67)
8	69	4.8 (5.34)	74	5.01 (4.59)
9	69	5.2 (5.61)	73	4.67 (4.15)
10	69	4.62 (4.71)	74	4.86 (4.54)

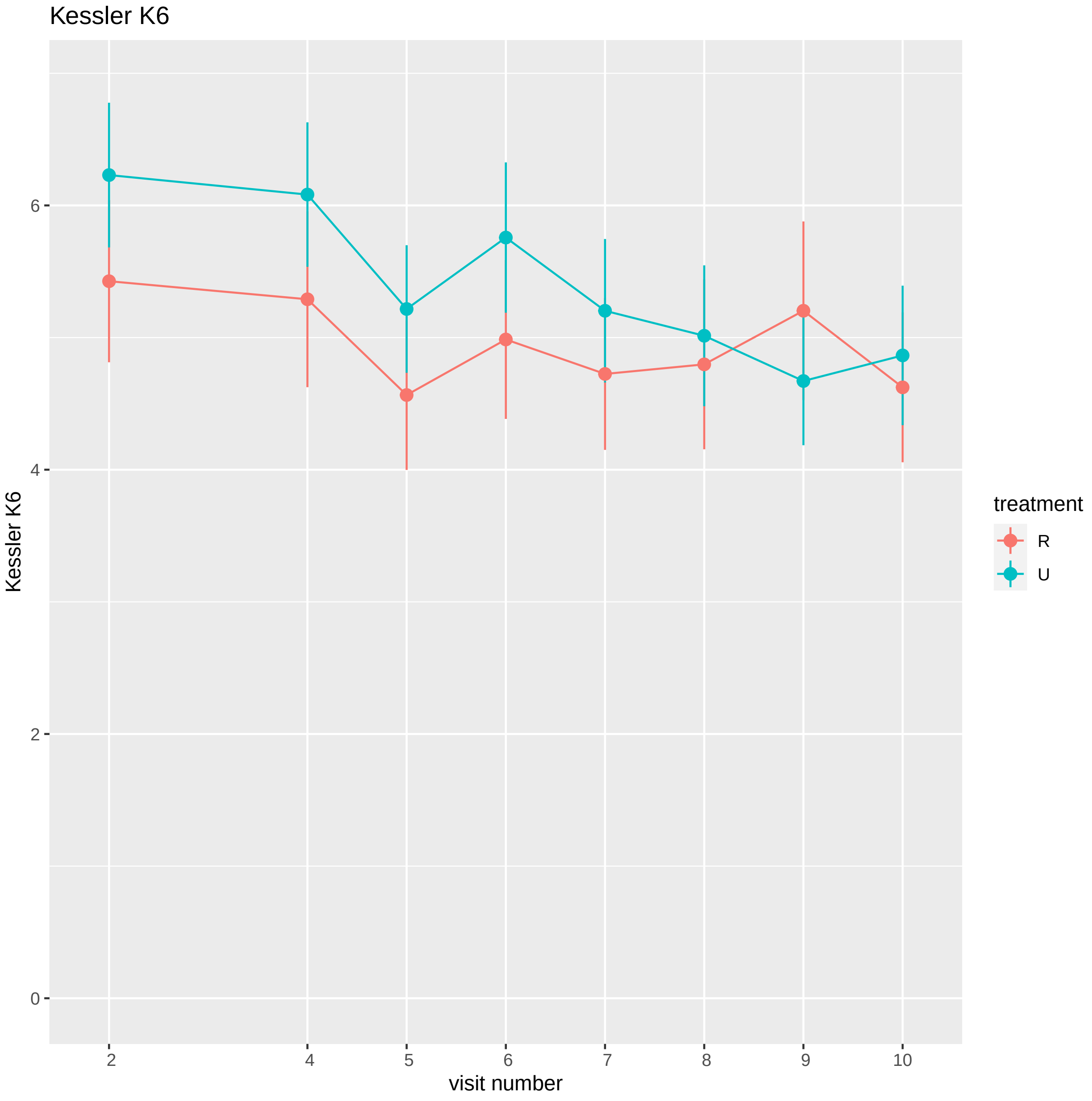

Psychiatry: Perceived Stress.

Visit	N for RNC	mean (SD) of PSS for RNC	N for UNC	mean (SD) of PSS for UNC
2	69	17.06 (7.84)	74	17.74 (7.91)
4	69	15.61 (7.83)	74	16.34 (8.16)
5	69	14.96 (7.74)	73	15.96 (7.67)
6	69	15.38 (7.63)	74	16.58 (8.04)
7	69	15.9 (7.95)	74	15.54 (8.01)
8	69	15.03 (7.91)	74	15.78 (8.03)
9	69	14.68 (8.44)	73	15.68 (6.95)
10	69	14.97 (7.57)	74	15.09 (7.83)

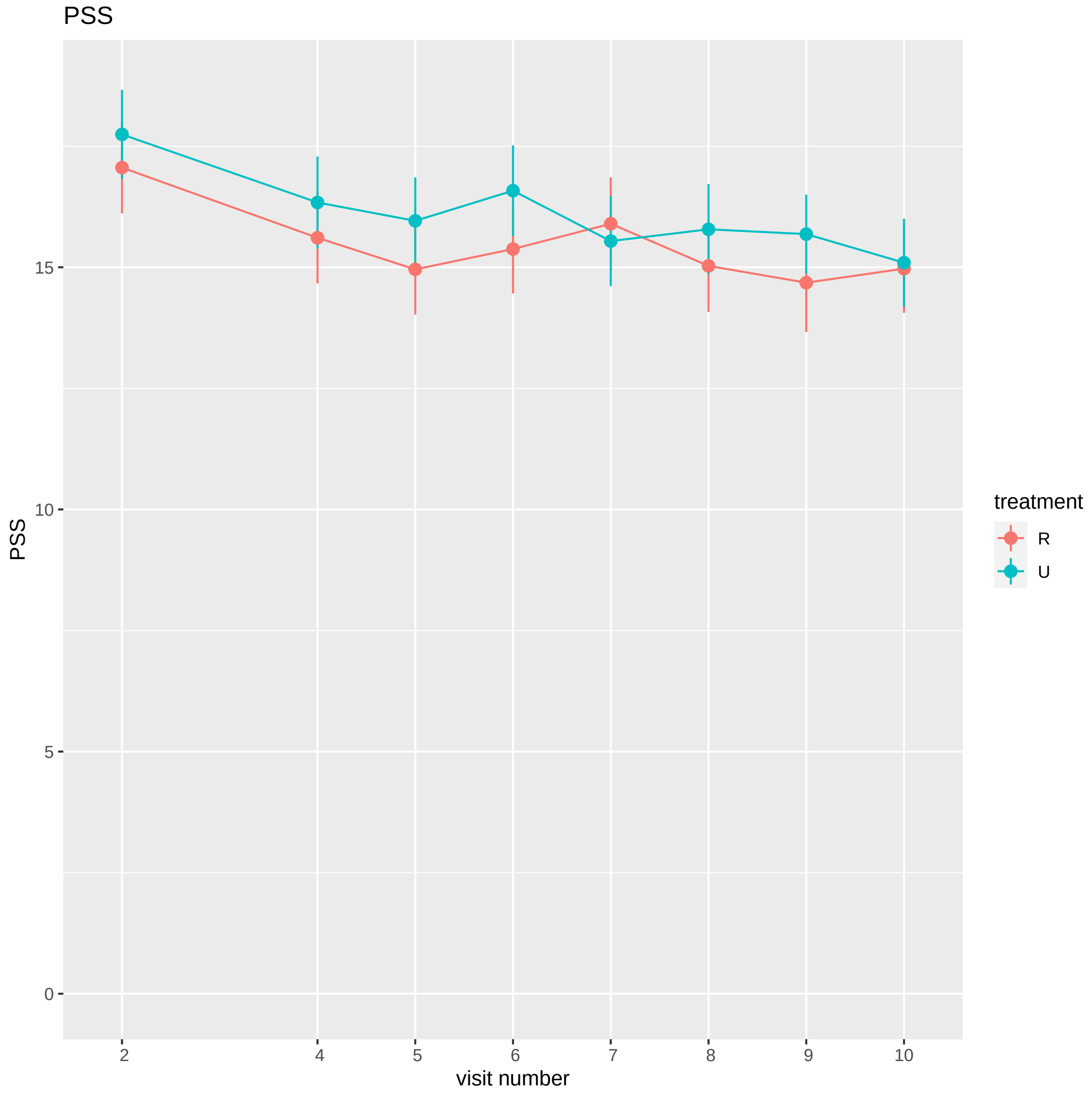

Psychiatry: CES-D.

Visit	N for RNC	mean (SD) of CES-D for RNC	N for UNC	mean (SD) of CES-D for UNC
7	69	17.07 (7.92)	71	17.48 (7.14)
10	69	17.3 (7.99)	73	16.34 (7.38)

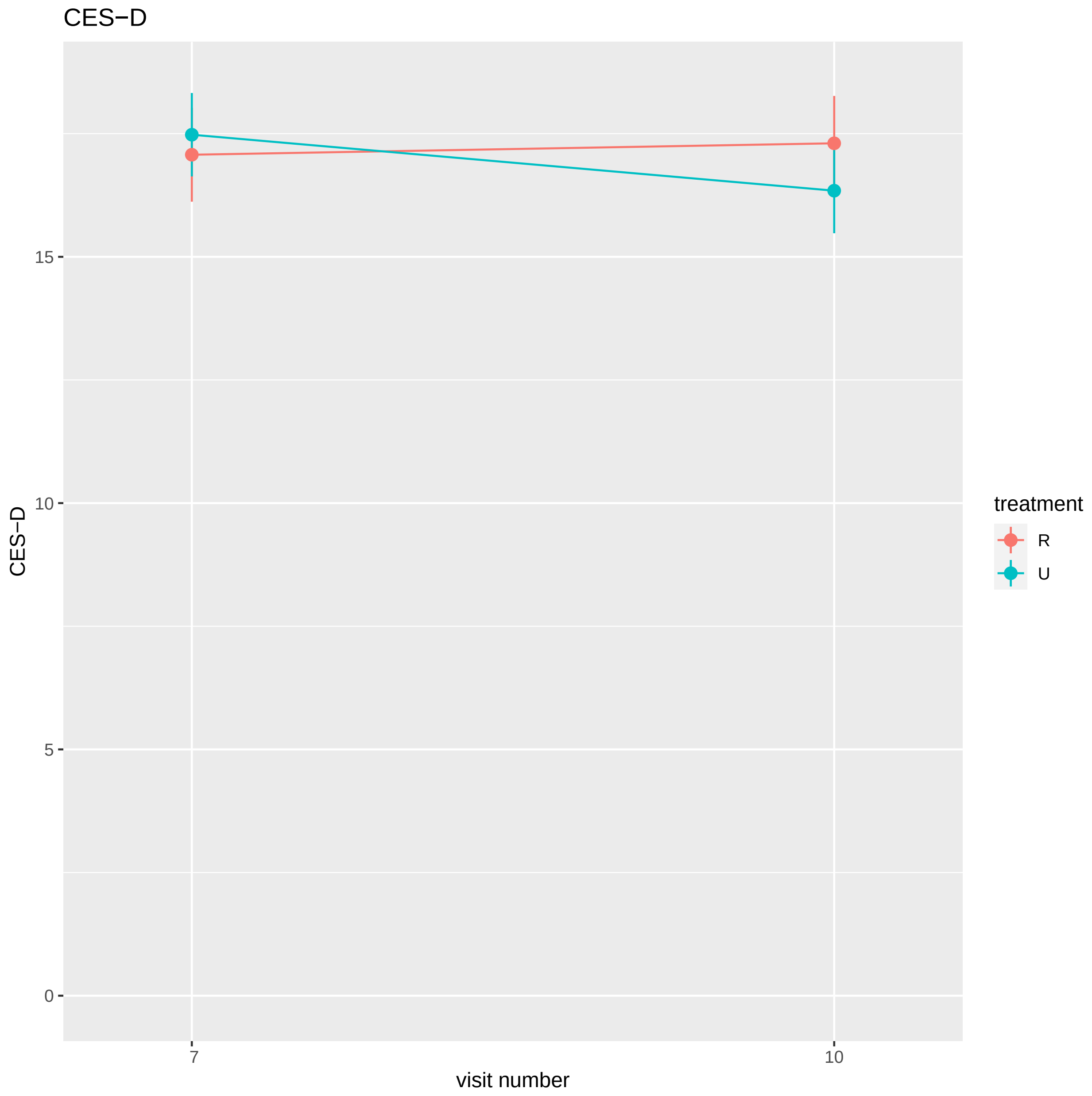

Adverse Health Effects: CCQ - COPD Total.

Visit	N for RNC	mean (SD) of Respiratory CCQ Total for RNC	N for UNC	mean (SD) of Respiratory CCQ Total for UNC
2	69	0.98 (0.85)	74	1.03 (0.73)
4	69	0.88 (0.79)	73	1.03 (0.77)
5	69	1.08 (0.94)	74	0.99 (0.8)
6	69	0.95 (0.92)	74	1.09 (0.82)
7	69	0.89 (0.83)	74	1.03 (0.84)
8	69	0.9 (0.85)	74	1.03 (0.79)
9	69	0.85 (0.84)	73	1.06 (0.85)
10	69	0.86 (0.9)	74	1.02 (0.8)

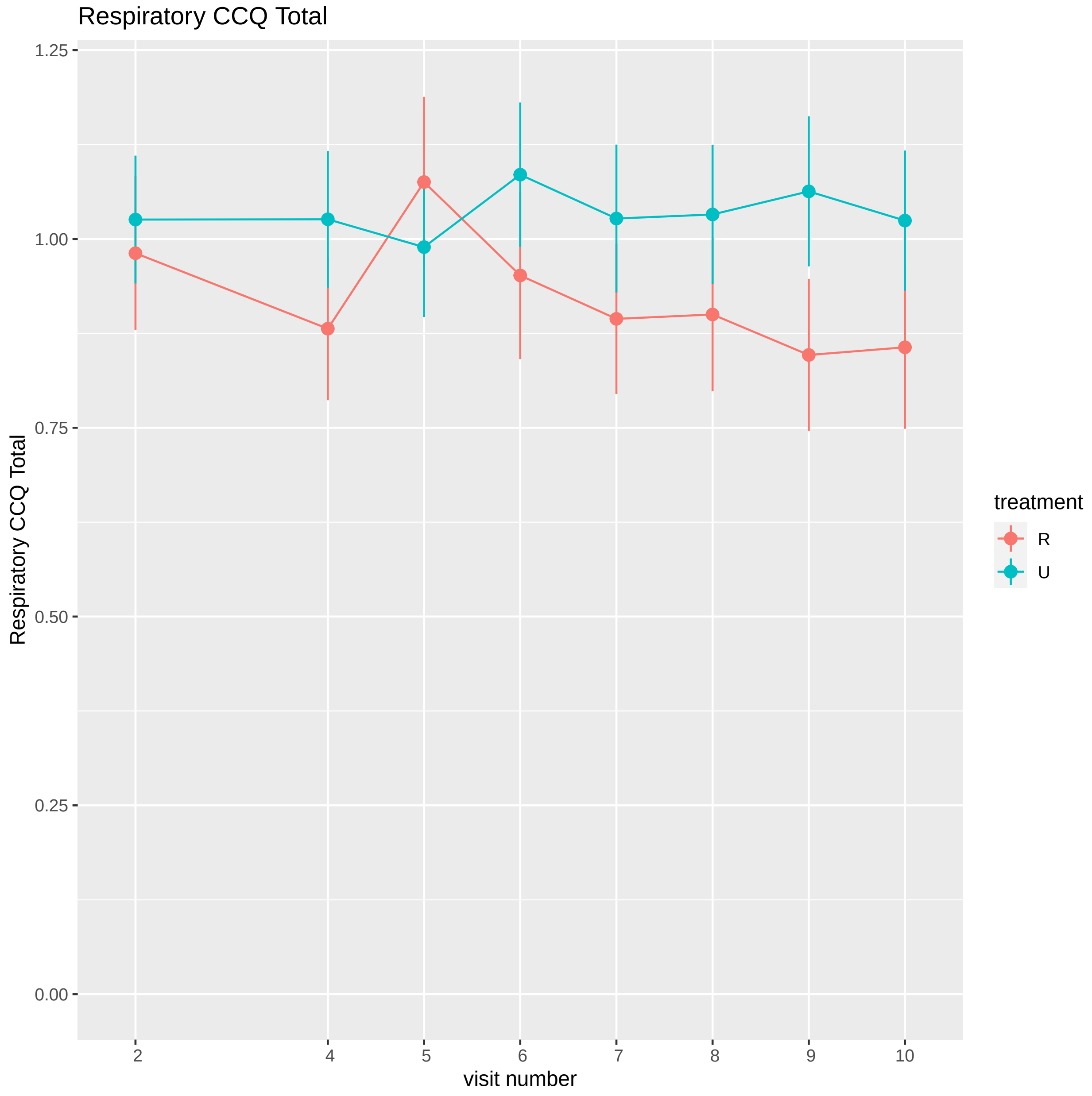

Adverse Health Effects: Systolic Blood Pressure.

Visit	N for RNC	mean (SD) of SBP for RNC	N for UNC	mean (SD) of SBP for UNC
2	69	124.36 (14.89)	74	123.27 (14.64)
3	69	123.67 (15.19)	74	122.73 (13.91)
4	69	121.84 (12.63)	74	121.46 (14.22)
5	69	121.96 (15.13)	74	121.12 (14.59)
6	69	123.91 (16.51)	74	123.38 (14.36)
7	69	121.39 (15.12)	74	123.93 (15.64)
8	69	122.91 (15.82)	74	122.03 (14.56)
9	69	120.84 (14.46)	73	123.4 (13.91)
10	69	122.84 (13.87)	73	123.44 (15.02)

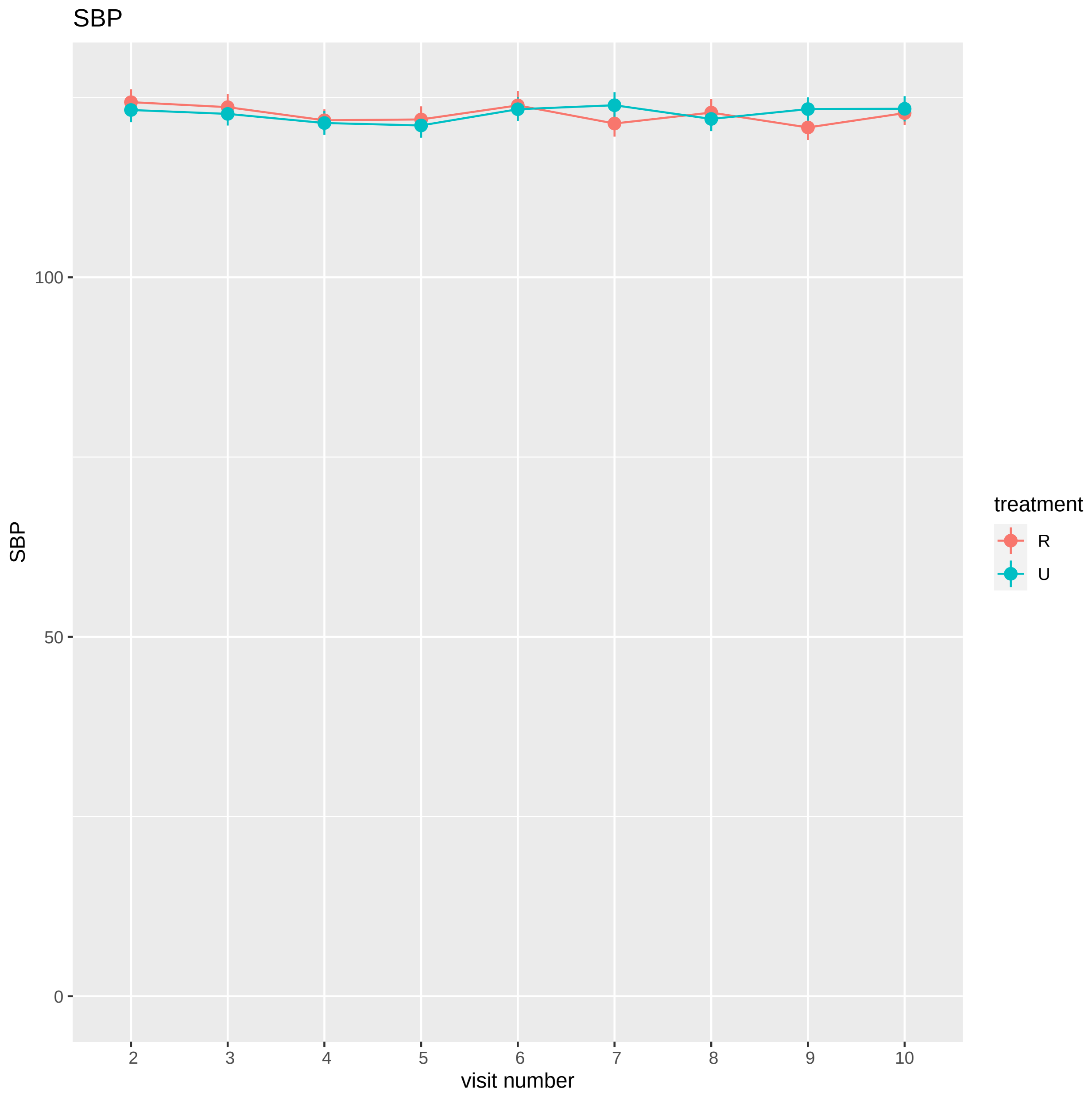

Adverse Health Effects: Diastolic Blood Pressure.

Visit	N for RNC	mean (SD) of DBP for RNC	N for UNC	mean (SD) of DBP for UNC
2	69	80.41 (10.4)	74	77.95 (10.23)
3	69	79.26 (10.49)	74	77.66 (10.37)
4	69	78.67 (9.8)	74	77.24 (9.62)
5	69	78.94 (10.09)	74	77.12 (10.49)
6	69	79.09 (12.04)	74	78.46 (10.61)
7	69	77.25 (10.85)	74	77.64 (11.68)
8	69	79.22 (11.38)	74	76.38 (11)
9	69	77.58 (10.66)	73	77.64 (10.92)
10	69	79.01 (12.24)	73	79.92 (16.25)

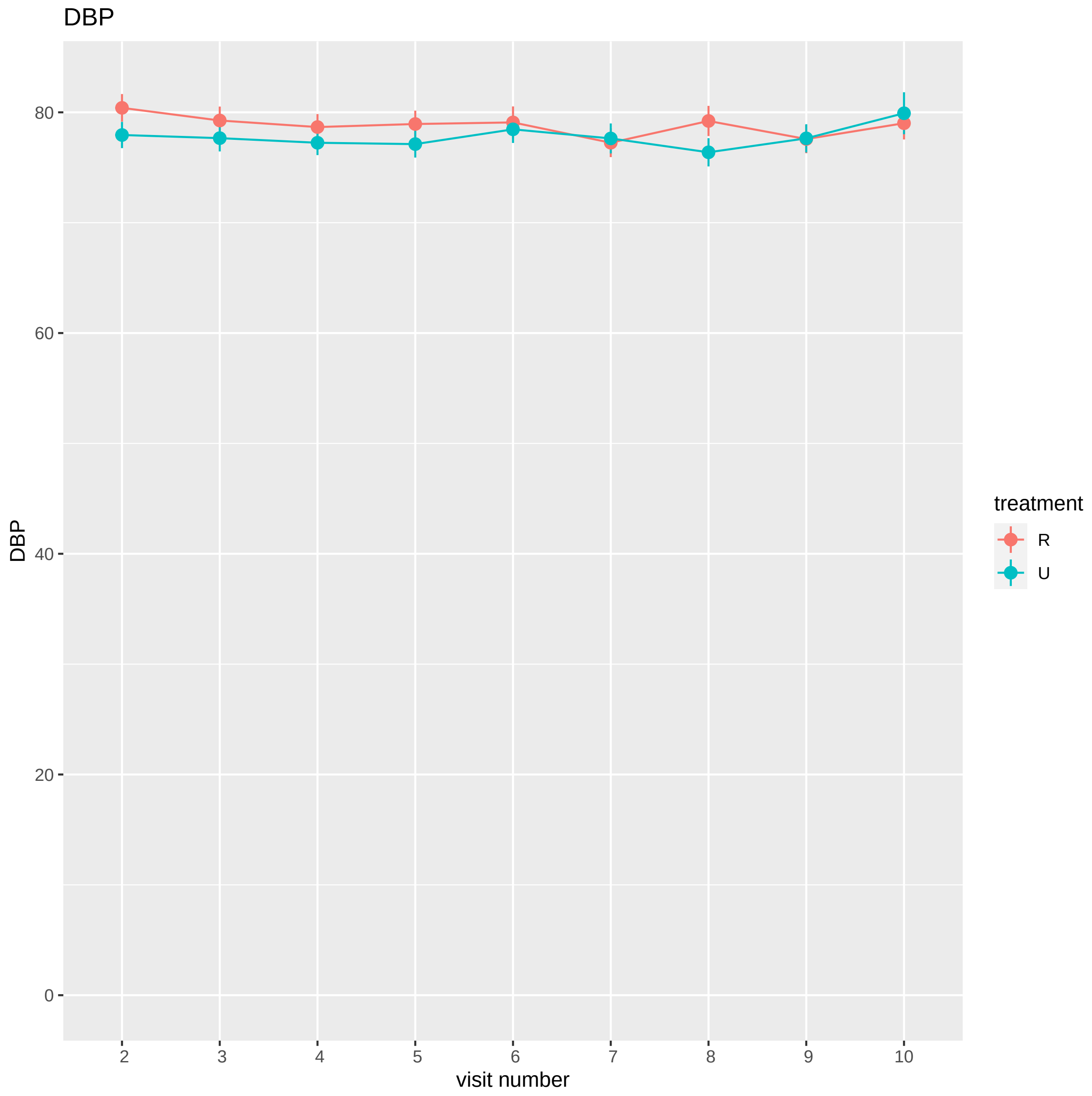

Pulse.

Visit	N for RNC	mean (SD) of Pulse for RNC	N for UNC	mean (SD) of Pulse for UNC
2	69	80.61 (12.54)	74	79.34 (13.22)
3	69	83.75 (12.16)	74	80.62 (12.33)
4	69	81.23 (13.73)	74	82.09 (12.42)
5	69	80.91 (12.02)	74	79.99 (13.01)
6	69	79.75 (13.08)	74	79.91 (14.62)
7	69	78.33 (11.17)	74	78.26 (10.81)
8	69	76.48 (14.57)	74	78.3 (13.23)
9	69	78.41 (11.57)	73	78.27 (13.35)
10	69	76.19 (12.2)	73	77.89 (12.34)

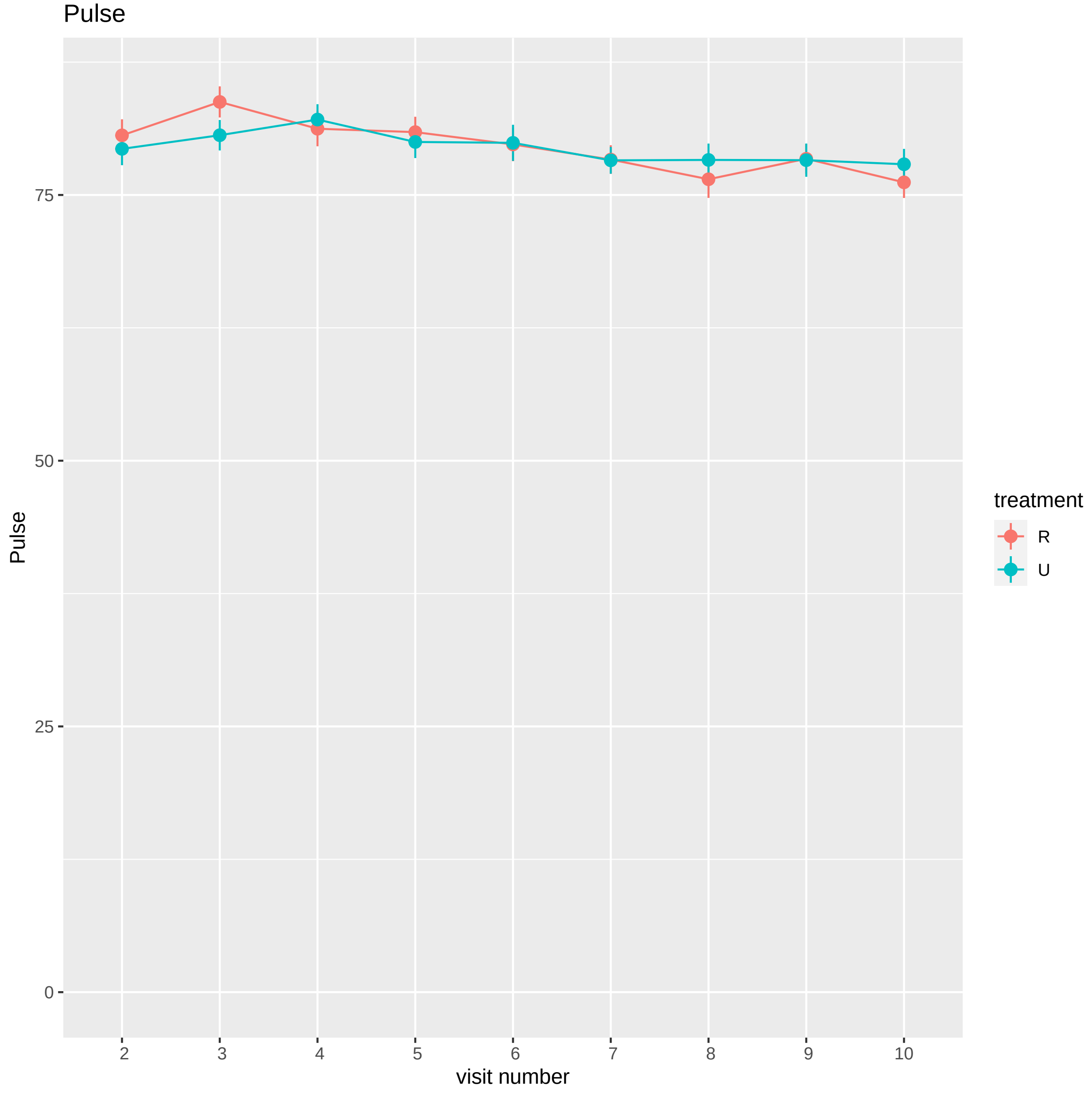

Weight.

Visit	N for RNC	mean (SD) of Weight for RNC	N for UNC	mean (SD) of Weight for UNC
2	69	196.36 (52.74)	74	190.51 (57.47)
3	69	196.8 (52.72)	74	192.52 (57.02)
4	69	198.35 (52.1)	74	192.48 (57.6)
5	68	195.39 (53.27)	74	192.09 (57.08)
6	69	197.48 (52.34)	74	191.79 (56.91)
7	69	198.41 (52.59)	74	191.71 (56.97)
8	69	198.32 (50.77)	74	191.11 (57.33)
9	69	199.15 (52.32)	73	193.08 (57.16)
10	69	198.08 (49.83)	73	192.73 (57.52)

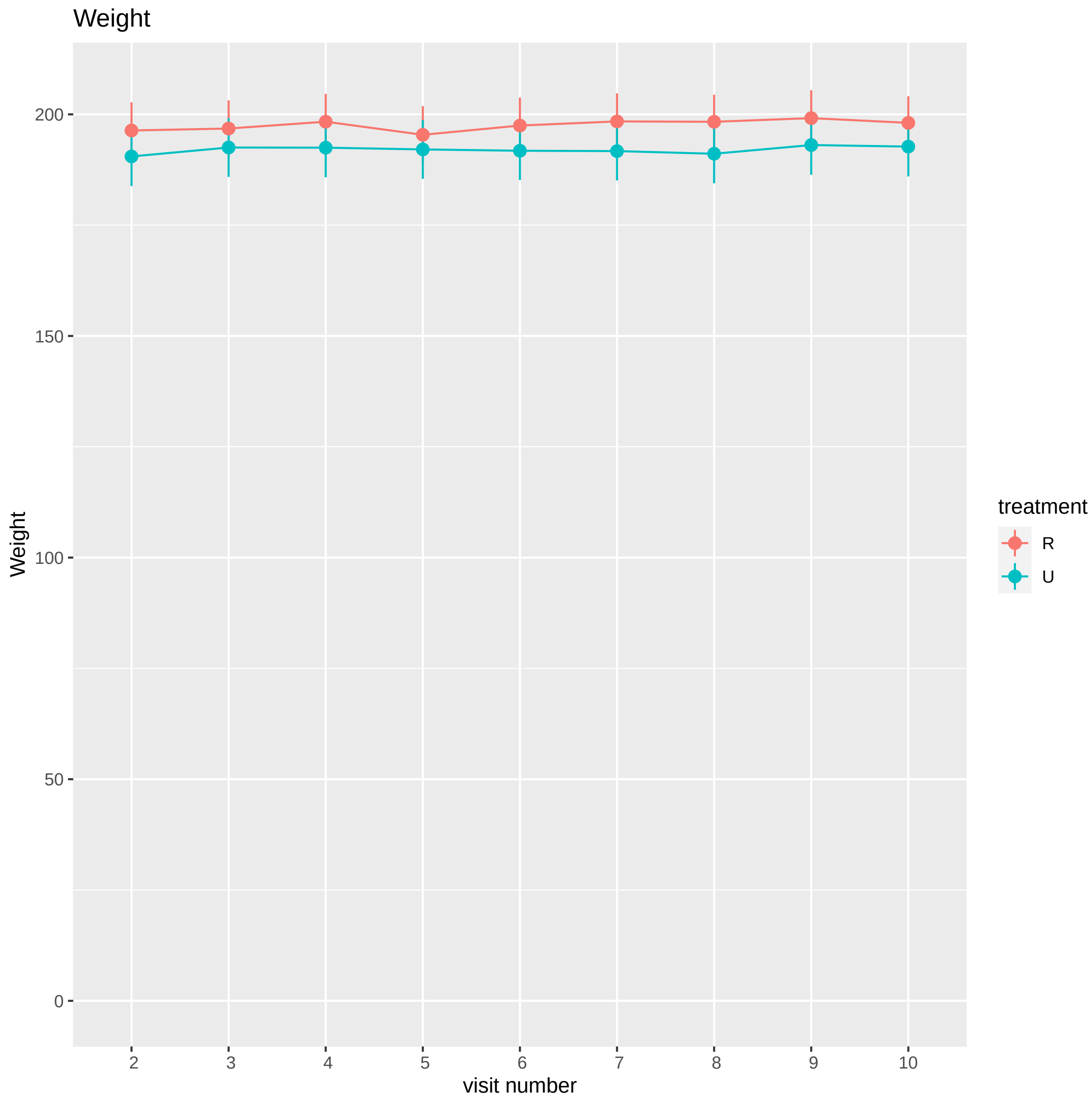

Questionnaire on Smoking Urges.

Visit	N for RNC	mean (SD) of QSU for RNC	N for UNC	mean (SD) of QSU for UNC
2	69	33.62 (15.94)	74	32.39 (15)
4	69	30.39 (15.47)	74	33.38 (15.69)
5	68	32.21 (14.72)	74	31.12 (14.81)
6	68	32.51 (15.41)	74	34.72 (16.63)
7	69	32.93 (15.4)	74	31.39 (14.81)
8	69	28.64 (14.49)	74	31.68 (15.73)
9	69	26.86 (15.5)	73	32.08 (15.97)
10	69	26.52 (15.53)	74	32.05 (14.81)

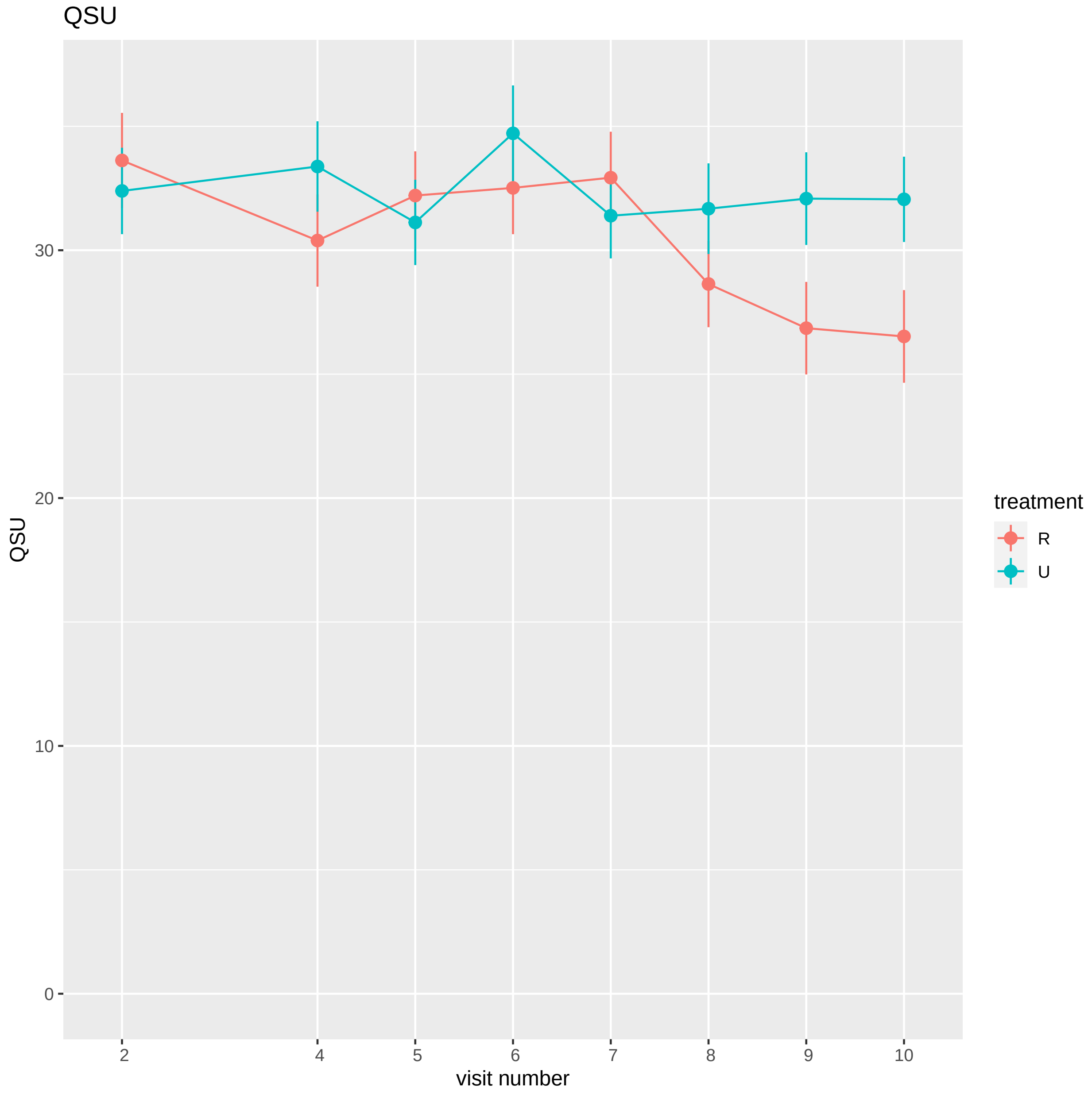

Adverse Health Effects: FEV1 Liters.

Visit	N for RNC	mean (SD) of FEV1 Liters for RNC	N for UNC	mean (SD) of FEV1 Liters for UNC
2	69	2.79 (0.78)	74	2.72 (0.69)
5	68	2.73 (0.78)	72	2.73 (0.7)
8	69	2.75 (0.76)	72	2.72 (0.71)
9	69	2.76 (0.76)	72	2.72 (0.71)
10	67	2.78 (0.77)	71	2.71 (0.69)

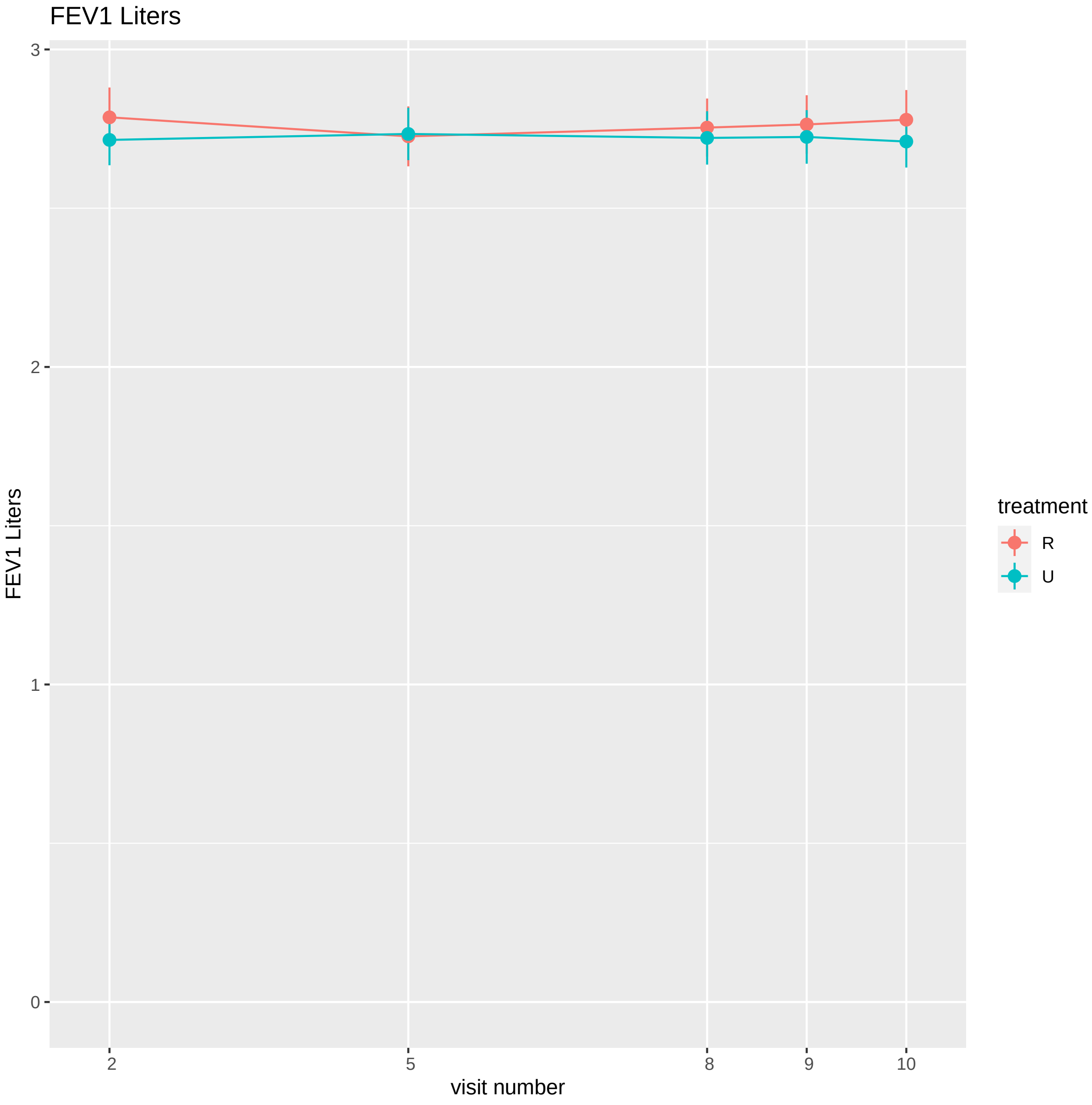

Biomarker: GSSP/GSH Ratio.

Visit	N for RNC	mean (SD) of GSSP/GSH Ratio for RNC	N for UNC	mean (SD) of GSSP/GSH Ratio for UNC
2	45	0.18 (0.08)	51	0.19 (0.08)
4	47	0.17 (0.07)	52	0.17 (0.08)
6	25	0.17 (0.05)	25	0.18 (0.05)
10	25	0.18 (0.06)	25	0.18 (0.05)

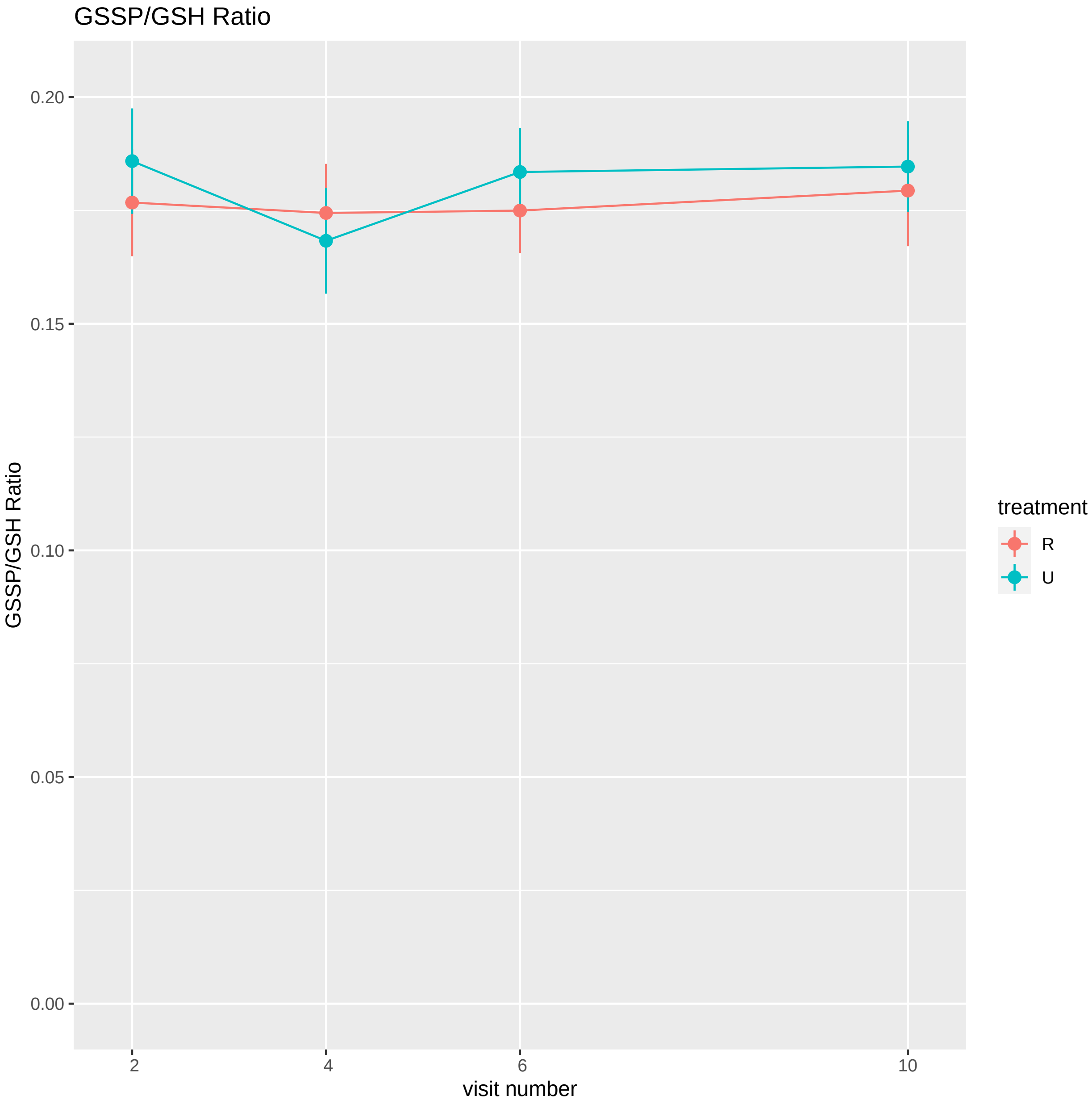

Biomarker: (Standardized) Total Urine 8-Isoprostanes (ng/mg creatinine).

Visit	N for RNC	mean (SD) of (Standardized) Total Urine 8-Isoprostanes (ng/mg creatinine) for RNC	N for UNC	mean (SD) of (Standardized) Total Urine 8-Isoprostanes (ng/mg creatinine) for UNC
4	25	4.27 (1.67)	25	3.82 (1.55)
6	25	3.92 (1.56)	25	4.45 (2.04)
10	25	3.98 (1.66)	25	4.25 (2.08)

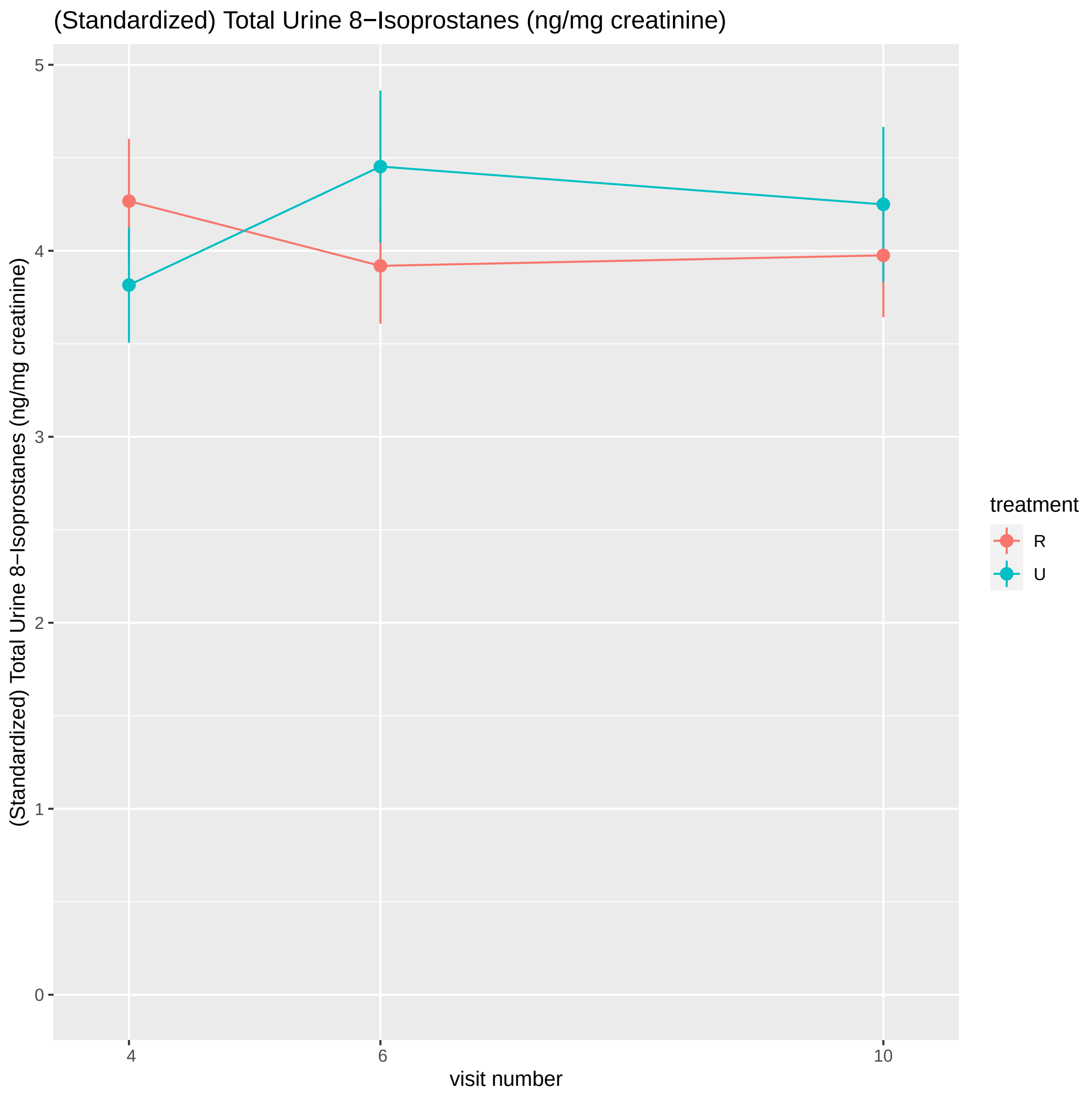

Biomarker: (Standardized) Total Urine NNAL (pmol/mg creatinine).

Visit	N for RNC	mean (SD) of (Standardized) Total Urine NNAL (pmol/mg creatinine) for RNC	N for UNC	mean (SD) of (Standardized) Total Urine NNAL (pmol/mg creatinine) for UNC
2	26	1.37 (1.03)	26	1.53 (1.06)
4	26	1 (0.74)	26	1.04 (0.7)
5	26	0.98 (0.69)	25	1.09 (0.73)
6	26	0.96 (1.18)	26	1.18 (0.66)
7	26	0.72 (0.65)	26	1.24 (0.96)
8	25	0.6 (0.67)	26	1.24 (0.78)
9	26	0.57 (0.78)	26	1.05 (0.7)
10	26	0.71 (1)	25	1.3 (1.08)

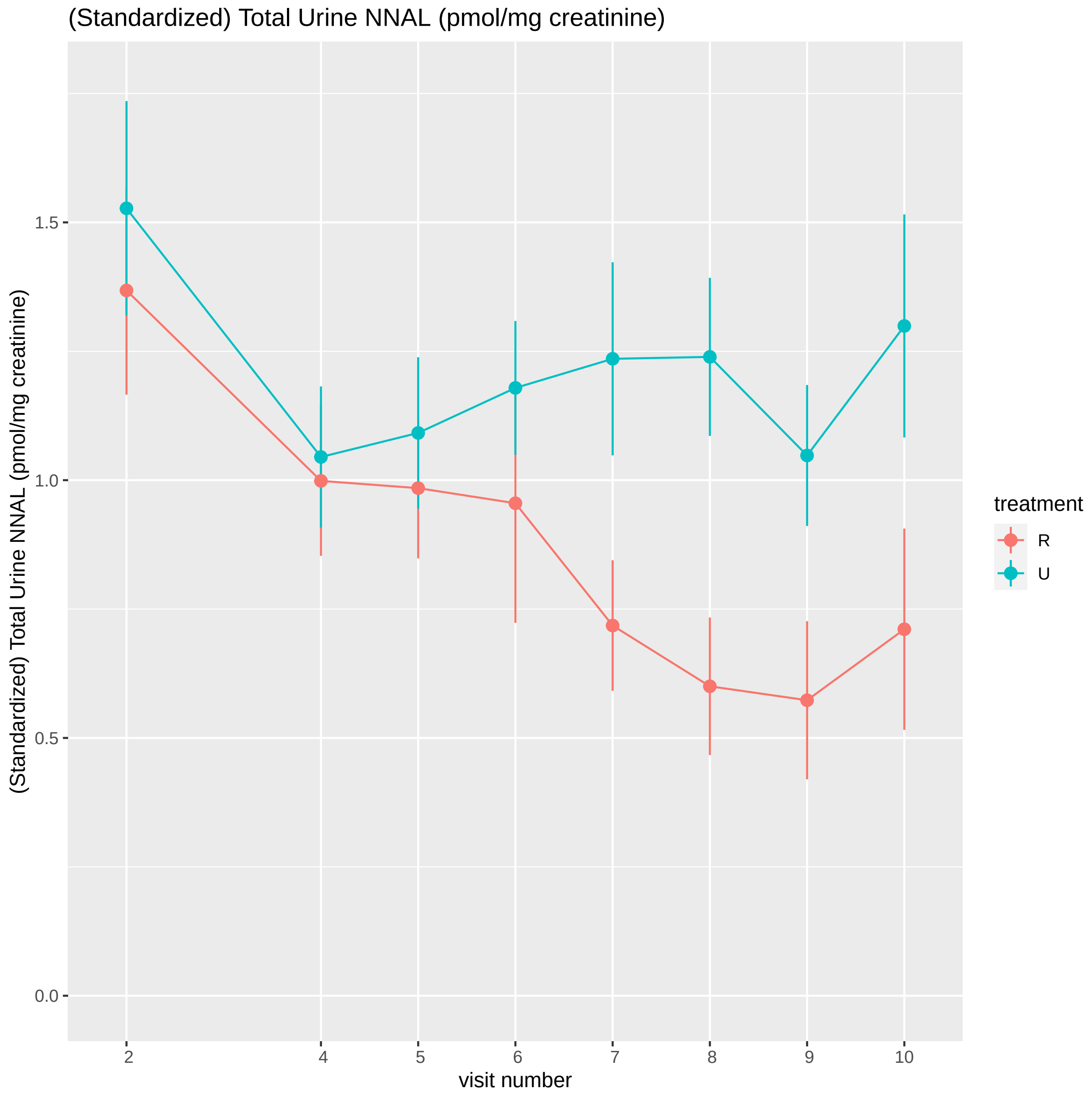

Alcohol consumption.

Visit	N for RNC	mean (SD) of Audit C for RNC	N for UNC	mean (SD) of Audit C for UNC
2	69	1.83 (2.22)	73	2.21 (2.39)
4	69	1.88 (2.32)	73	2.18 (2.36)
6	69	1.86 (2.36)	74	2.18 (2.62)
8	68	1.82 (2.3)	74	2.09 (2.52)
10	69	1.8 (2.23)	74	2.05 (2.29)

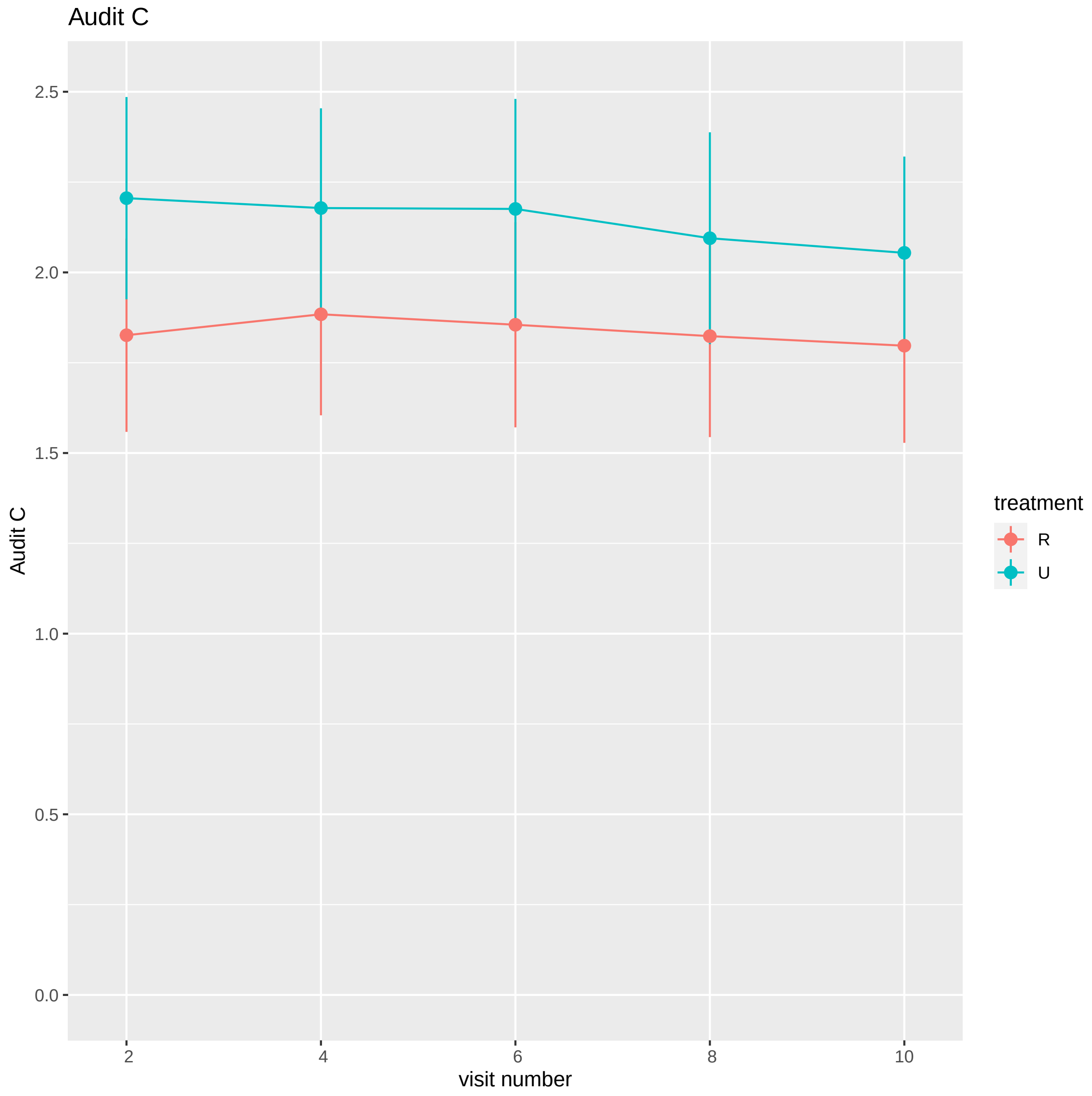

This is the end of the report.
